# Anxiolytic and Anticonvulsant Effects of *Moringa* Isothiocyanate-1 (MIC-1) in Adult Zebrafish

**DOI:** 10.3390/biology15171523

**Published:** 2026-09-03

**Authors:** João Victor Alves Martins, José Ednésio da Cruz Freire, Maria Rayane Correia de Oliveira, Gabriela Alves do Nascimento, Lia Gomes Crisóstomo Sabóia, Paulo Adenes Teixeira Coelho, Lorena Silva Lima, Keciany Alves de Oliveira, Francisco Lucas Alves Batista, Hamilton Mitsugu Ishiki, Antonio Eufrásio Vieira-Neto, Sacha Aubrey Alves Rodrigues Santos, Adriana Rolim Campos, Lucas Soares Frota, Selene Maia de Morais, Renalison Farias-Pereira, Francisco Ernani Alves Magalhães, Ariclécio Cunha de Oliveira

**Affiliations:** 1 Postgraduate Program in Nutrition and Health, Health Sciences Center, Laboratory of Endocrine Physiology and Metabolism, State University of Ceará, Fortaleza 60741-000, Ceará, Brazil; joaovictornut@gmail.com (J.V.A.M.); keciany.oliveira@uece.br (K.A.d.O.); 2 Biochemistry and Gene Expression Laboratory, Superior Institute of Biomedical Sciences, State University of Ceará, Fortaleza 60714-903, Ceará, Brazil; jednesio@gmail.com; 3 Postgraduate Program in Nutrition and Health, NutriFisher Study Group, Laboratory of Bioprospection of Natural Products and Biotechnology, Fortaleza 60660-000, Ceará, Brazil; rayane.correia@uece.br (M.R.C.d.O.); gabi.nascimento@aluno.uece.br (G.A.d.N.); lia.gomes@aluno.uece.br (L.G.C.S.); paulo.adenes@aluno.uece.br (P.A.T.C.); nutricionistalorenalima@gmail.com (L.S.L.); luccas.batista@uece.br (F.L.A.B.); hamilton.ishiki@uece.br (H.M.I.); ernani.magalhaes@uece.br (F.E.A.M.); 4 Postgraduate Program in Biotechnology, Northeast Biotechnology Network, Experimental Biology Nucleus, University of Fortaleza, Fortaleza 60811-650, Ceará, Brazil; aevneto@gmail.com (A.E.V.-N.); sachaaubrey@hotmail.com (S.A.A.R.S.); adrirolim@unifor.br (A.R.C.); 5 Postgraduate Program in Natural Sciences, Center for Science and Technology, Laboratory of Chromatographic and Spectroscopic Analyses, State University of Ceará, Fortaleza 60714-903, Ceará, Brazil; lucassfrota@gmail.com (L.S.F.); selenemaiademorais@gmail.com (S.M.d.M.); 6 Department of Biological Sciences, Kean University, Kean Union Campus, Union, NJ 07083, USA; rfariasp@kean.edu

**Keywords:** *Moringa oleifera*, anticonvulsant, anxiolytic, GABAergic and serotonergic modulation

## Abstract

The ‘miracle tree’ *Moringa oleifera* from the Moringaceae family is recognized for its rich nutritional value and therapeutic properties. This study aimed to evaluate the phytopharmaceutical compound *Moringa* isothiocyanate-1 (MIC-1) isolated from hydroalcoholic extract of *M. oleifera* seeds for the treatment of seizures and anxiety in adult zebrafish. MIC-1 (up to 0.5 mg/mL) did not show any acute toxic effects over a 96-h period. Moreover, MIC-1 (0.005 and 0.05 mg/mL) delayed the onset of seizures induced by pentylenetetrazol, indicating anticonvulsant activity. The anticonvulsant effects of MIC-1 (0.05 mg/mL) were reversed by flumazenil, suggesting a mechanism involving the GABA_A_ receptor. Furthermore, MIC-1 (0.05 mg/mL) has anxiolytic effects, with involvement of the serotonergic and GABAergic systems. In silico assays confirmed the interaction of MIC-1 with the GABA_A_, 5-HT_1B_, 5-HT_2A/C_, and 5-HT_3A/B_ receptors. Additionally, MIC-1 was effective in treating anxiety related to alcohol withdrawal in zebrafish. These findings suggest the pharmacological potential of MIC-1 for the development of new treatments for seizures and anxiety.

## Simple Summary

Anxiety and seizures are common neurological conditions that can greatly affect quality of life, and current medicines may cause unwanted effects such as drowsiness, sedation, and dependence. Therefore, new treatment options are needed. This study investigated a natural compound obtained from *Moringa oleifera* seeds, called *Moringa* isothiocyanate-1 (MIC-1), using adult zebrafish, a small laboratory fish commonly used in biomedical research. MIC-1, up to 0.5 mg/mL, did not cause acute toxic effects and did not impair normal movement. MIC-1 delayed the onset of experimentally induced seizures and reduced anxiety-like behavior, including anxiety caused by alcohol withdrawal. These findings suggest that MIC-1’s effects may involve brain systems responsible for controlling neuronal inhibition and mood. Computer-based analyses supported the possible interaction between MIC-1 and neurotransmitter receptors. Overall, these results indicate that MIC-1 may be a promising bioactive compound for developing new approaches to manage anxiety and seizures. However, further studies are required to confirm its safety, effectiveness, and possible use in humans.

## 1. Introduction

Anxiety is a mental health condition where an excessive and persistent worry impairs the ability to control thoughts. Common symptoms include muscle tension, sleep disturbances, irritability, and fatigue [1]. The COVID-19 pandemic not only impacted physical health but also had a profound effect on the mental well-being of the global population. The rise in anxiety cases, driven by social isolation, uncertainty about the future, and disruptions to daily life, underscores the urgent need for further research and the development of effective strategies for the prevention and treatment of this disorder [2].

Anxiety and stress can influence neuronal excitability, increasing the likelihood of abnormal electrical discharges in the brain. In individuals with a predisposition to seizures, these factors can act as triggers, potentially leading to epileptic seizures. Furthermore, the experience of recurrent seizures can itself induce anxiety and fear, creating a vicious cycle that perpetuates and exacerbates the condition [3].

The primary treatment for anxiety and seizures typically involves the use of benzodiazepines. Although benzodiazepines are effective in symptom control, they are associated with side effects such as drowsiness, sedation, and sleep disturbances, negatively impacting life quality [4,5,6]. As a result, there has been growing interest in identifying natural alternatives to benzodiazepines that offer similar therapeutic benefits with a lower risk of side effects and dependence. Several medicinal plants and bioactive compounds have demonstrated anxiolytic and sedative properties, often acting through neurobiological mechanisms like those targeted by benzodiazepines. These natural agents present a safer and potentially more effective therapeutic option for managing anxiety disorders, contributing to an improved quality of life for patients. Moreover, combining natural therapies with conventional pharmacological treatments may enhance therapeutic outcomes and reduce adverse effects, supporting a more personalized and integrative approach to treatment [7,8].

*Moringa* isothiocyanate-1 (MIC-1) is a secondary metabolite derived from the hydrolysis of glucosinolates by the enzyme myrosinase, found predominantly in *M. oleifera* seeds. Extraction using polar solvents, such as water and ethanol, optimizes the process yield, producing an extract rich in MIC-1 (with concentrations ranging from 35% to 45%) from which the compound can be purified [9,10]. MIC-1 exhibits biological activities comparable to those of other natural isothiocyanates, including anticancer, anti-inflammatory, antioxidant, neuroprotective, and antimicrobial effects. This pharmacological profile is directly linked to its ability to modulate antioxidant and anti-inflammatory molecular pathways [11,12,13]. This is particularly significant given that oxidative stress and inflammation are interdependent pathophysiological mechanisms in epilepsy, with their interaction promoting the onset, maintenance, and propagation of epileptic seizures [14,15]. Therefore, MIC-1 is a bioactive compound with a promising role in cellular protection including within the central nervous system.

Given the urgent need for anticonvulsant and anxiolytic agents that offer greater safety and a lower incidence of side effects, this study aimed to evaluate the therapeutic potential of the compound MIC-1 in the treatment of seizures and anxiety in adult zebrafish (*Danio rerio*) models.

## 2. Materials and Methods

### 2.1. Drugs and Reagents

The following materials were used in this study: ultrapure water, commercial ethanol (96%), ethyl alcohol (99.5%; Êxodo Científica^®^, Sumaré, São Paulo, Brazil), pentylenetetrazol (PTZ; Sigma-Aldrich^®^, St. Louis, MO, USA), cyproheptadine (CYPRO; Sigma-Aldrich^®^, St. Louis, MO, USA), pizotifen (PIZ; Sigma-Aldrich^®^, St. Louis, MO, USA), granisetron (GSTN; Sigma-Aldrich^®^, St. Louis, MO, USA), diazepam (DZP; Neo Química^®^, Anápolis, Goiás, Brazil), flumazenil (Fmz; Sandoz^®^, Basel, Switzerland), dimethyl sulfoxide (DMSO; Dinâmica Química^®^, Indaiatuba, São Paulo, Brazil), and fluoxetine (FLX; Eli Lilly^®^, Indianapolis, IN, USA). Yellow sugarcane spirit (ACAA; Ypióca^®^, Maranguape, Ceará, Brazil) containing ethanol (38% EtOH) was used following the protocol described by Ferreira and collaborators [16].

### 2.2. Preparation of the Hydroalcoholic Extract and MIC-1

MIC-1 was extracted and isolated as previously described [9,12]. First, moringa seed extract was obtained using ground seeds incubated in water at a 1:3 ratio for 2 h at 37 °C. These steps were performed following the optimized protocol, as previously described, to obtain the maximum yield of isothiocyanate [9]. Subsequently, ethanol was added to facilitate the extraction of isothiocyanates, followed by filtration, rotary evaporation, and freeze-drying. Purification of MIC-1 was carried out using the freeze-dried moringa seed extract via fraction centrifuge liquid chromatography [17]. The extract was resuspended in ethanol (200 mg/mL), sonicated for 30 min, filtered through a 0.2 μm membrane, and injected into an FCPC^®^ 1000 instrument (Kromaton, Angers, France), operated using version 1.0 of the associated control software. MIC-1 was eluted using a solvent mixture of hexane, methanol, water, and ethyl acetate, collected, dried, and lyophilized. The final purified compound (purity > 99%) was stored at −20 °C. Its identity was subsequently confirmed by liquid chromatography–mass spectrometry (LC-MS) using a Q Exactive Plus ESI-Orbitrap mass spectrometer coupled to a Dionex Ultimate 3000 HPLC-UV system (Thermo Scientific, Waltham, MA, USA), by comparing its retention time, UV spectrum, and mass spectrometric data with those of previously prepared standards [9].

### 2.3. Zebrafish

Adult wild-type zebrafish (*D. rerio*) were purchased from Agroquímica LTDA (Fortaleza, Ceará, Brazil) with the following parameters: both sexes, age of 60–90 days old, length of 3.5 ± 0.5 cm; weight of 0.4 ± 0.1 g. Before the experimental procedures, 50 fish were housed in an aquarium (40 × 20 × 25 cm) with chlorine-free water and ProtecPlus^®^ (Labcon/Alcon^®^, Camboriú, Santa Catarina, Brazil) under a 14:10 h light/dark cycle for 24 h. The aquaria were provided with continuous aeration using air pumps and equipped with submerged filtration systems. Throughout the acclimation period, water quality was kept at 25 °C and pH 7.0. Zebrafish had free access to Spirulina^®^ (Labcon/Alcon^®^, Camboriú, Santa Catarina, Brazil) feed until 24 h before the beginning of the experiments. Animal care and protocols were approved by the institutional Animal Use Ethics Committee of the State University of Ceará (CEUA-UECE; No. 04009489/2023).

### 2.4. General Treatment

Different concentrations and treatments of MIC-1, vehicle controls, and positive controls were given orally (20 µL; p.o) to adult zebrafish (n = 6/group). The concentrations for MIC-1 ranged from 0.005 to 0.5 mg/mL (10-fold serial dilutions), whereas for diazepam (DZP; positive control), it was 10 mg/mL, and the vehicle control consisted of 3% DMSO. An additional naïve group, which received no treatment, were included as a baseline as indicated in the respective figures.

### 2.5. Non-Clinical Safety Evaluation

#### 2.5.1. Acute Toxicity (96 h)

The acute toxicity protocol was performed as previously described by Batista et al., [18] according with the OECD guidelines [19]. The treatments were orally given to the animals, which were subsequently kept at rest. Animals were observed over a 96-h period. No deaths were observed due to MIC-1 for all tested concentrations, indicating that the lethal concentration (LC_50_) values for MIC-1 are likely to be greater than 0.5 mg/mL.

#### 2.5.2. Locomotor Activity (Open Field Test)

After one-hour treatment, an individual fish was put in a glass Petri dish (10 × 1.5 cm), marked with four quadrants, with 60 mL of aquarium water from where the fish were kept. The number of line crossings was counted over a 5-min period as a measure of zebrafish locomotor activity [20].

### 2.6. Anticonvulsant Activity (Pentylenetetrazol Test)

The anticonvulsant activity of MIC-1 was determined by measuring the time spent with pentylenetetrazol (PTZ)-induced convulsive-like behavior (i.e., the clonus-like seizures with a loss of posture) [21]. After one-hour treatment, animals were individually exposed to 250 mL of 30 mM PTZ.

We also tested the involvement of the GABAergic system using the same test with animals pretreated with flumazenil (Fmz; a GABA_A_ receptor antagonist) [21]. Zebrafish were first pre-treated intraperitoneally (i.p.) with 20 µL of 0.1 mg/mL Fmz. Fifteen minutes later, zebrafish received treatment with MIC-1, DZP, or their respective vehicles for one hour before PTZ exposure.

### 2.7. Anxiolytic-like Effect (Light & Dark Test)

After one-hour treatment, animals were individually placed in a test aquarium with light and dark zones. Then, the time spent in the light zone over a 5-min period was used as a parameter to predict the anxiolytic effects of treatments as previously established [22].

We tested the involvement of the serotonergic (5-HT) system by using receptor antagonists and treatments before placing zebrafish in a light/dark test aquarium [23]. Initially, zebrafish were treated with MIC-1 (0.005 mg/mL; 20 µL; p.o.), fluoxetine (FLX; 1.25 × 10^−3^ mg/mL; 20 µL; p.o.; anxiolytic control), cyproheptadine (CYPRO; 5-HT_2A/B_ antagonist; 0.8 mg/mL; 20 µL; p.o.), pizotifen (PIZ; 5-HT_1B_ and 5-HT_2A/C_ antagonist; 0.8 mg/mL; 20 µL; p.o.), or granisetron (GSTN; 5-HT_3A/B_ antagonist; 0.5 mg/mL; 20 µL; p.o.). In a subsequent experiment, zebrafish were pre-treated orally with cyproheptadine, pizotifen, or granisetron, 15 min prior to the administration of MIC-1 or FLX. Control groups included animals treated with the vehicle (3% DMSO) and a naive group (untreated).

Similarly, we used the light/dark test to evaluate the potential neuromodulation of MIC-1 via the GABAergic system. Animals were pre-treated with flumazenil (Fmz; 0.1 mg/mL; 20 µL; i.p.) 15 min before receiving 0.005 mg/mL MIC-1 or 10 mg/mL DZP. The behavior of each fish was assessed in the light–dark test 30 or 60 min after treatment with Fmz or MIC-1/DZP, respectively [23].

### 2.8. Anxiety Induced by Alcohol Withdrawal (Light & Dark Test)

Since MIC-1 (0.005–0.5 mg/mL) demonstrated anxiolytic effects via the GABAergic system, we evaluated MIC-1’s potential for treating anxiety induced by alcohol withdrawal [16]. Yellow sugarcane spirit (ACAA) was used as a source of ethanol at a concentration of 38% (*v*/*v*), which is non-toxic to zebrafish. Animals received daily ACAA (days 1-5; first phase) followed by a withdrawal period over the subsequent five days (days 6-10; second phase). On day 11 (third phase), zebrafish received the treatment with MIC-1 or DZP. The time spent in the light zone by the animals was measured daily in the light & dark test following a one-hour treatment [22].

### 2.9. Statistical Analysis

The data are expressed as the mean ± standard error of the mean (S.E.M.) for each group of six animals. After confirming the normality of data distribution and the homogeneity of variances, differences between groups were analyzed using one-way analysis of variance (ANOVA), followed by Tukey’s post hoc test. All statistical analyses were performed using GraphPad Prism v. 8.4.3 software. Statistical significance was set at *p* < 0.05.

### 2.10. Molecular Docking

The interactions between the receptors (GABA_A_, 5-HT_1B_, 5-HT_2A/C_, and 5HT_3A/B_) and the ligands (MIC-1 and positive controls: DZP and FLX) were analyzed In silico using molecular docking simulations. The minimized 3D structures of the ligands, MIC-1 (modeled via ChemSketch, version 2025), DZP, and FLX, were obtained from the PubChem database (CIDs: 3016, 3386). The three-dimensional structures of the GABA_A_ receptor (human β3-homopentamer) [24] and the 5-HT_1B_, 5-HT_2A/C_, and 5-HT_3A/B_ receptors were retrieved from the Protein Data Bank (PDB) under the IDs: 4COF, 5V54, 6BQH, and 6W1Y, respectively. These crystal structures have resolutions ranging from 2.30 to 2.97 Å.

Ligands were prepared using the LigPrep protocol with the Epik tool (Schrödinger Release 2026-3, Schrödinger, LLC, New York, NY, USA), as documented previously [25]. Molecular docking was performed using HEX software, version 8.0.0 [26], which automatically adjusted docking poses based on interaction energies between the ligands and all possible receptor surface sites. The resulting clusters were analyzed and visualized in detail using PyMol version 1.4.7.

## 3. Results

### 3.1. MIC-1 Did Not Change Locomotor Activity

**Figure 1:**
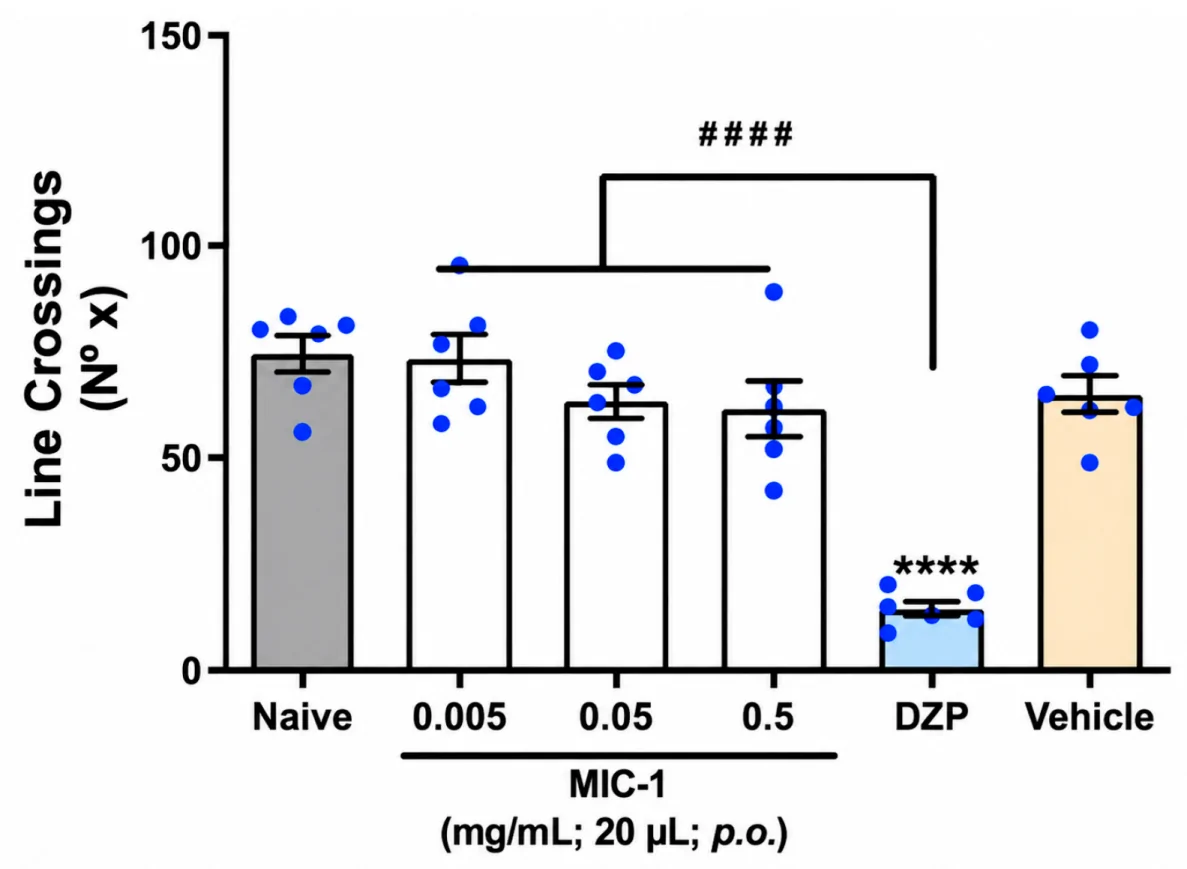
*Moringa* isothiocyanate-1 (MIC-1) did not change the locomotor activity of adult zebrafish. MIC-1, 3% DMSO (vehicle) or diazepam (DZP; 10 mg/mL) were given orally to the animals (20 µL; p.o.) one hour before the open field test (0–5 min). Mean ± standard error of the mean (SEM). N = 6/group. One-way ANOVA followed by Tukey’s post hoc test (**** *p* < 0.0001 vs. Naive or Vehicle; ^####^ *p* < 0.0001 vs. DZP).

MIC-1, up to 0.5 mg/mL, did not exhibit acute toxicity nor sedative effect, as MIC-1 did not cause death nor alter the locomotor activity of adult zebrafish (*D. rerio*). On the other hand, diazepam, a positive sedative control, reduced zebrafish locomotor activity (*q* = 12.85, *p* > 0.0001 vs. Naive; *q* = 10.81, *p* > 0.0001 vs. Vehicle), (F_5,30_ = 22.80), as shown in Figure 1.

### 3.2. Anticonvulsant Activity of MIC-1 via GABAergic System

**Figure 2:**
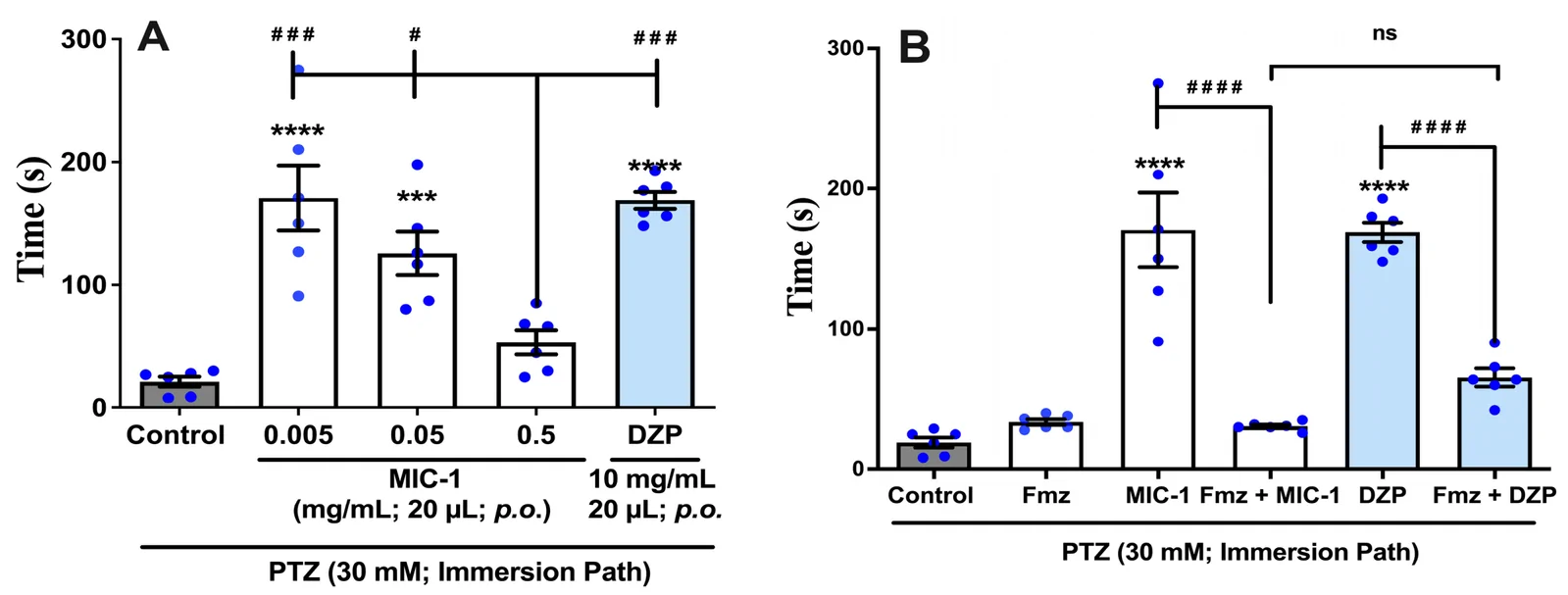
Anticonvulsant activity of *Moringa* isothiocyanate-1 (MIC-1) via the GABAergic system. MIC-1, 3% DMSO (control), or 10 mg/mL diazepam (DZP) were orally given to adult zebrafish (20 µL; p.o.) before immersion in a 30 mM of pentylenetetrazol (PTZ) solution. Panel (**A**): Effect of MIC-1 on PTZ-induced convulsive behavior in adult zebrafish. Panel (**B**): The flumazenil (Fmz)-reversed effects of MIC-1 and DZP’s anticonvulsant activities. Pre-treatment with 0.1 mg/mL Fmz (20 µL; i.p.) was administered before treatments with 0.05 mg/mL MIC-1 and 10 mg/mL DZP. Mean ± standard error of the mean (SEM); n = 6 animals/group. Statistical analysis was performed using one-way ANOVA followed by Tukey’s post hoc test. *** *p* < 0.001 and **** *p* < 0.0001 compared with the Control group; ^#^ *p* < 0.05, ^###^ *p* < 0.001, and ^####^ *p* < 0.0001 compared with the MIC-1 or DZP groups. ns, not significant (*p* > 0.05).

MIC-1 exhibited a significant anticonvulsant effect in zebrafish (F_4,25_ = 19.71), as evidenced by a marked increase in the latency to the onset of clonus-like seizures with loss of posture. MIC-1 at 0.005 and 0.05 mg/mL increased latency compared to the control (*q* = 9.763, *p* < 0.0001 and *q* = 6.824, *p* < 0.001, respectively). The effect of MIC-1 was comparable to that of DZP (*q* = 9.643, *p* < 0.0001 vs. control), with no significant difference between the MIC-1 and DZP-treated groups (*q* = 0.1197 and *q* = 2.819, *p* > 0.05), as shown in Figure 2A.

To evaluate whether the anticonvulsant effect of 0.05 mg/mL MIC-1 involved the GABAergic system, we pre-treated the animals with 0.1 mg/mL flumazenil (Fmz; a GABA_A_ receptor antagonist). The MIC-1’s anticonvulsant effect was significantly reversed by Fmz, as indicated by a marked reduction in seizure latency (*q* = 12.03, *p* < 0.0001; MIC-1 vs. Fmz + MIC-1). Similarly, DZP’s anticonvulsant effect was also significantly reversed by Fmz (*q* = 8.878, *p* < 0.0001; DZP vs. Fmz + DZP), with no difference between the flumazenil-reversed effects of MIC-1 and DZP (*q* = 2.993, *p* > 0.05; Fmz + MIC-1 vs. Fmz + DZP), (F_5,30_ = 36.30), as shown in Figure 2B. These findings indicate that the anticonvulsant effects of MIC-1 are likely mediated through GABA_A_ receptor activation, as evidenced by their reversal upon flumazenil pre-treatment.

### 3.3. Anxiolytic-like Effect of MIC-1

**Figure 3:**
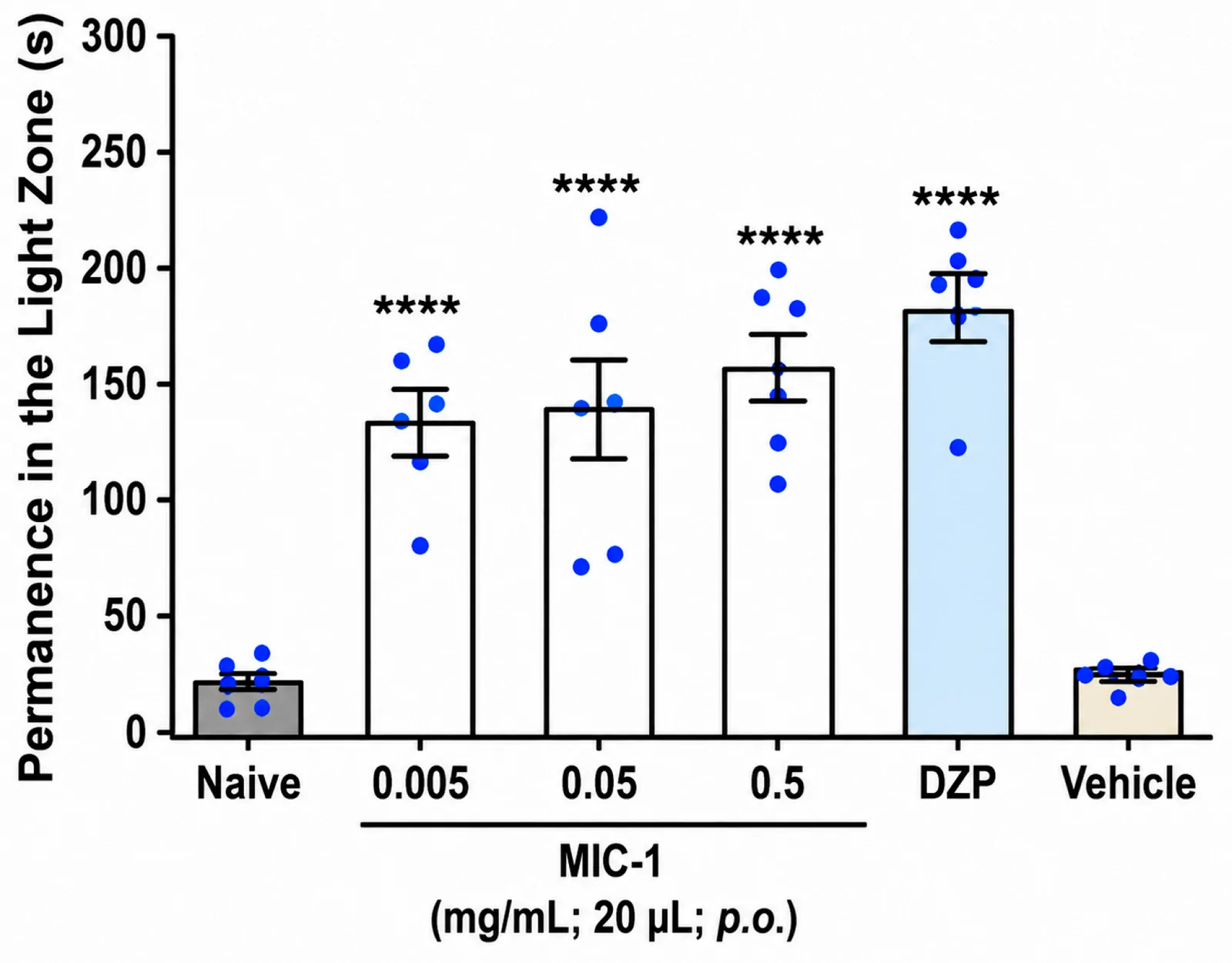
Anxiolytic-like effect of *Moringa* isothiocyanate-1 (MIC-1). Adult zebrafish received oral (20 µL; p.o.) treatments of MIC-1 or diazepam (DZP; 10 mg/mL), or their vehicle controls (3% DMSO) one hour before the light & dark test. The time spent in the light zone for 5 min was measured. An untreated group (naïve) was also concomitant evaluated. Mean ± standard error of the mean (SEM). n = 6/group. One-way ANOVA followed by Tukey’s post hoc test: (**** *p* < 0.0001 vs. Naive or Vehicle).

MIC-1, at concentrations of 0.005, 0.05, and 0.5 mg/mL, significantly increased the time spent by the animals in the light zone compared to the naive group (*q* = 7.655, 8.168, and 9.240, respectively; *p* < 0.0001) and the vehicle group (*q* = 7.690, 8.203, and 9.275, respectively; *p* < 0.0001). The anxiolytic effect of MIC-1 was statistically similar to that of diazepam (DZP; 10 mg/mL; 20 µL; p.o.) at all concentrations tested (*q* = 3.286, 2.773, and 1.701, respectively; *p* > 0.05). Diazepam significantly increased the time spent in the light zone compared to both the naive (*q* = 10.94, *p* < 0.0001) and vehicle (*q* = 10.98, *p* < 0.0001) groups (F_5,30_ = 22.96), as shown in Figure 3.

### 3.4. Neuromodulation via the Serotonergic 5-HT_2A/C_ System

**Figure 4:**
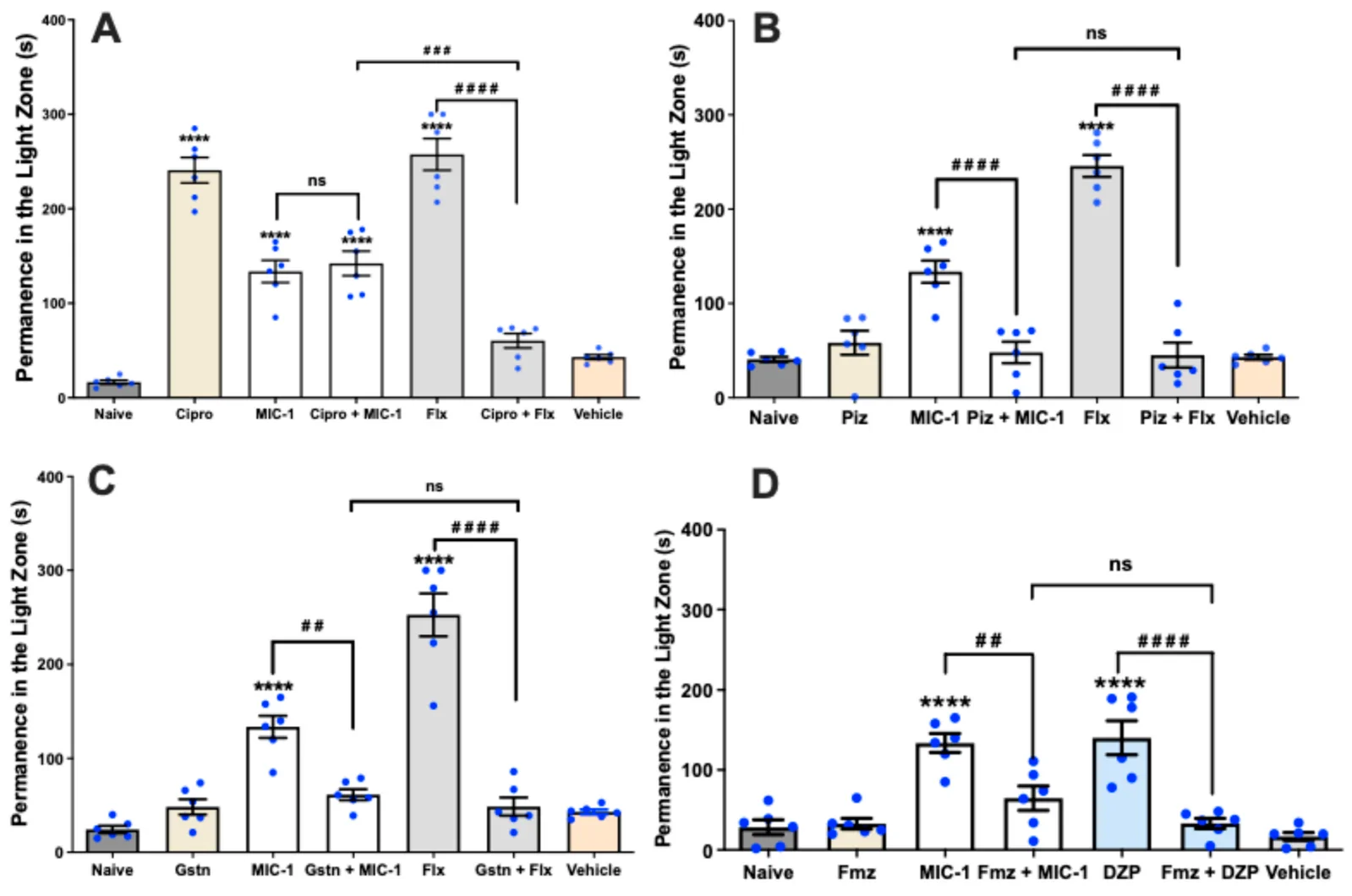
Effect of cyproheptadine (CIPRO) (Panel (**A**)), pizotifen (PIZ) (Panel (**B**)), granisetron (GSTN) (Panel (**C**)) and flumazenil (Fmz) (Panel (**D**)) on the anxiolytic-like effect of MIC-1 (0.005 mg/mL; 20 µL; p.o.) in adult zebrafish in the light & dark test (0–5 min). Naive—untreated animals. FLX—fluoxetine (anxiolytic control; 1.25 × 10^−3^ mg/mL; p.o.). DZP: diazepam (anxiolytic control; 10 mg/mL; 20 µL; p.o.). Vehicle—ultrapure water or 3% DMSO (20 µL; p.o.). Mean ± standard error of the mean (SEM). n = 6/group. One-way ANOVA followed by Tukey’s post hoc test was used for statistical analysis. In all panels, **** *p* < 0.0001 compared with the Naive or Vehicle groups. In Panel (**A**), ^###^ *p* < 0.001 compared with the CYPRO + MIC-1 group, and ^####^ *p* < 0.0001 compared with the FLX group. In Panel (**B**), ^####^ *p* < 0.0001 compared with the MIC-1 or FLX groups. In Panel (**C**), ^##^ *p* < 0.01 compared with the MIC-1 group, and ^####^ *p* < 0.0001 compared with the FLX group. In Panel (**D**), ^##^ *p* < 0.01 compared with the MIC-1 group, and ^####^ *p* < 0.0001 compared with the DZP group. ns, not significant.

The anxiolytic effect of MIC-1 (0.005 mg/mL; 20 µL; p.o.) was not significantly reversed by cyproheptadine (CYPRO; 0.8 mg/mL; 20 µL; p.o.), a 5-HT_2A/C_ antagonist [6] (*q* = 0.7763; *p* > 0.05; MIC-1 vs. CYPRO + MIC-1). In contrast, the anxiolytic effect of fluoxetine (FLX; 1.25 × 10^−3^ mg/mL; 20 µL; p.o.) was significantly reversed by cyproheptadine (*q* = 18.01; *p* < 0.0001; FLX vs. CYPRO + FLX), (F_6,35_ = 75.03), as shown in Figure 4A. These results suggest that the anxiolytic-like effects of MIC-1 are independent on the serotonergic 5-HT_2A/C_ receptor.

### 3.5. Neuromodulation via the Serotonergic 5-HT_1B_ and 5-HT_2A/2C_ Systems

The anxiolytic effect of MIC-1 (0.005 mg/mL; 20 µL; p.o.) was not significantly reversed by pizotifen (PIZ; 0.8 mg/mL; 20 µL; p.o.), a 5-HT_1B_ and 5-HT_2A/C_ antagonist [23] (*q* = 0.2738; *p* > 0.05; PIZ + MIC-1 vs. PIZ + FLX). In contrast, the anxiolytic effect of fluoxetine (FLX; 1.25 × 10^−3^ mg/mL; 20 µL; p.o.) was significantly reversed by pizotifen (*q* = 19.39; *p* < 0.0001; FLX vs. PIZ + FLX), (F_6,35_ = 55.36), Figure 4B. These results suggest that the anxiolytic effect of MIC-1 may not involve the serotonergic 5-HT_1B_ and 5-HT_2A/C_ receptors.

### 3.6. Neuromodulation via the Serotonergic 5-HT_3A/3B_ System

The anxiolytic effect of MIC-1 (0.005 mg/mL; 20 µL; p.o.) was significantly reversed by granisetron (GSTN; 0.5 mg/mL; 20 µL; p.o.), a 5-HT_3A/B_ receptor antagonist (*q* = 6.484; *p* < 0.01; MIC-1 vs. GSTN + MIC-1). The effect of fluoxetine (FLX; 1.25 × 10^−3^ mg/mL; 20 µL; p.o.) was also significantly reversed by granisetron (*q* = 18.27; *p* < 0.0001; FLX vs. GSTN + FLX), (F_6,35_ = 52.30), Figure 4C. These results suggest that the anxiolytic effect of MIC-1 is mediated, at least in part, by the serotonergic 5-HT_3A/B_ receptors.

### 3.7. Neuromodulation via the GABAergic System

The anxiolytic effect of MIC-1 (0.005 mg/mL; 20 µL; p.o.) was significantly reversed by flumazenil (Fmz; 0.1 mg/mL; 20 µL; i.p.), a GABA_A_ receptor antagonist (*q* = 5.736; *p* < 0.01; MIC-1 vs. Fmz + MIC-1). This reversal effect was statistically similar to that observed with diazepam (DZP; anxiolytic control; 10 mg/mL; 20 µL; p.o.), with no significant difference between the Fmz + MIC-1 and Fmz + DZP groups (*q* = 2.619; *p* > 0.05). Moreover, the anxiolytic effect of diazepam was also significantly reversed by flumazenil (*q* = 8.895; *p* < 0.0001; DZP vs. Fmz + DZP), (F_6,35_ = 18.51), Figure 4D. These results suggest that the anxiolytic-like effects of MIC-1 are dependent on the GABA_A_ receptor.

### 3.8. Treatment of Alcohol Withdrawal-Induced Anxiety with MIC-1

**Figure 5:**
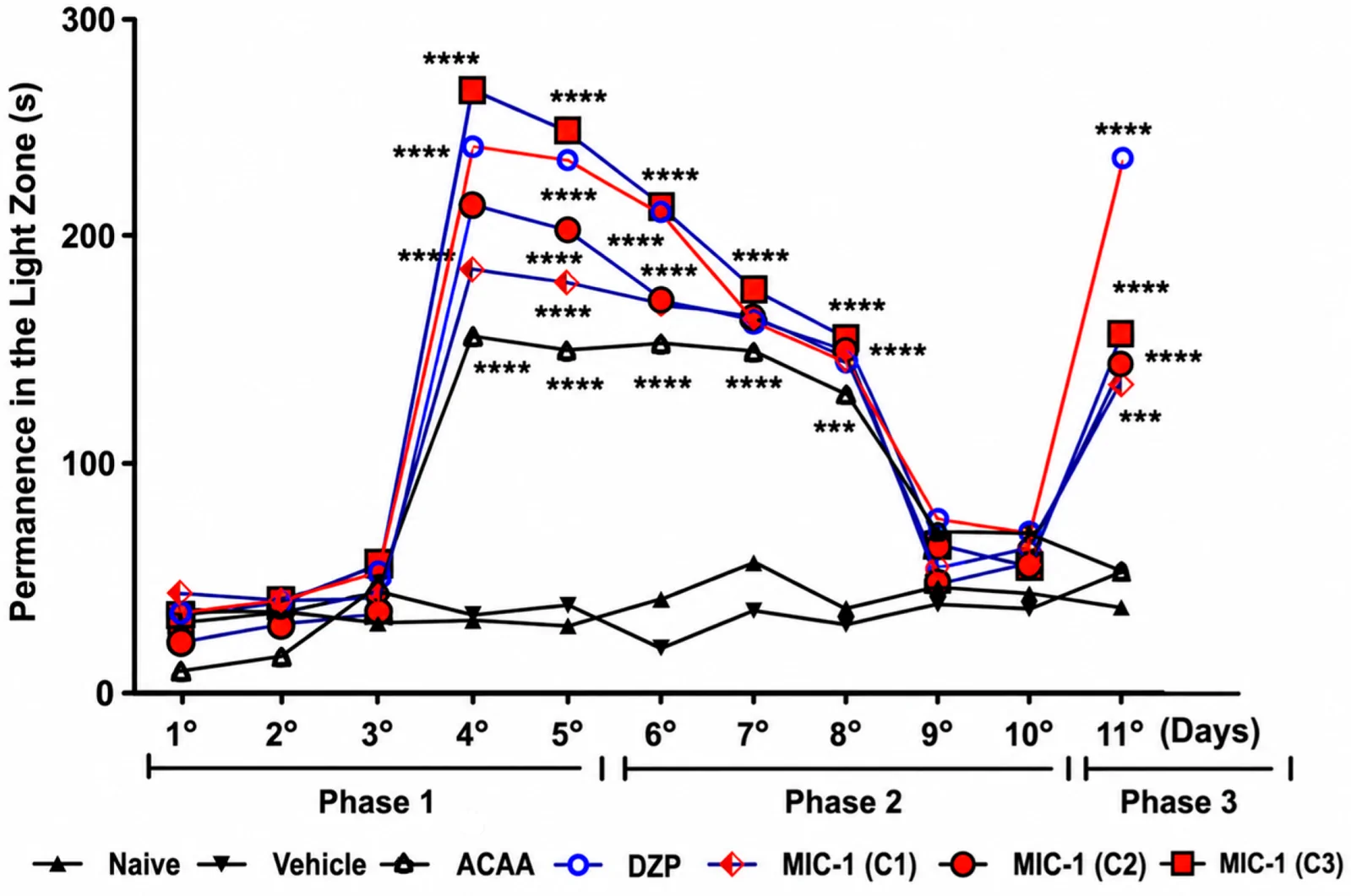
Effect of oral administration of MIC-1 (B) (20 μL; p.o.) on anxiety-like behavior in adult zebrafish induced by alcohol withdrawal. Groups were divided as: 1, naive (no treatment); 2, vehicle control; 3, ACAA (yellow sugarcane spirit); 4, ACAA + 10 mg/mL diazepam (DZP) on day 11; 5, ACAA + 0.005 mg/mL MIC-1 (C1); 6, ACAA + 0.05 mg/mL MIC-1 (C2); 7, ACAA + 0.5 mg/mL MIC-1 (C3). Treatments and ACAA (38% EtOH) were given orally (20 μL; p.o.). Vehicles were ultrapure water or 3% DMSO for ACAA or treatments, respectively. Mean ± standard error of the mean (SEM). n = 6/group. Two-way ANOVA followed by Tukey’s post hoc test (*** *p* < 0.001, **** *p* < 0.0001 vs. Naive group).

Zebrafish treated with ACAA (38% EtOH; *v*/*v*; 20 µL; p.o.), Groups 3–7, from days 4 to 8, showed a significant increase in the time spent in the light zone in the light & dark test compared to the control groups, Naive and Vehicle (3% DMSO). Alcohol withdrawal induced by ACAA caused a decrease in time spent in the light zone only on days 9 (*q* = 1.558, *p* > 0.05 vs. Naive; *q* = 2.041, *p* > 0.05 vs. Vehicle) and 10 (*q* = 1.644, *p* > 0.05 vs. Naive; *q* = 2.084, *p* > 0.05 vs. Vehicle), as shown in Figure 5 (F6,385 = 80.19). On the 11th day of alcohol withdrawal, anxiety was treated with diazepam (DZP; Group 4) or MIC-1 at 0.005, 0.05, or 0.5 mg/mL (20 µL; p.o.), Groups 5–7. As observed previously [Figure 3 (F_5,30_ = 22.96)], all MIC-1 concentrations effectively reduced anxiety induced by alcohol withdrawal, significantly increasing time spent in the light zone (*q* = 6.329, *p* > 0.001; *q* = 6.930, *p* > 0.0001; *q* = 7.704, *p* > 0.0001 vs. Naive; and *q* = 5.340, *p* > 0.01; *q* = 5.942, *p* > 0.001; *q* = 6.715, *p* > 0.0001 vs. Vehicle). However, the anxiolytic effect of MIC-1 at all tested doses was significantly different from that of diazepam (*q* = 6.511, *p* > 0.001; *q* = 5.910, *p* > 0.001; *q* = 5.136, *p* > 0.01 vs. DZP), which showed a stronger effect (*q* = 12.84, *p* > 0.0001 vs. Naive; *q* = 11.85, *p* > 0.0001 vs. Vehicle) as the anxiolytic control (10 mg/mL; 20 µL; p.o.) (F_6,385_ = 80.19), Figure 5. These results show that MIC-1 (Figure 5) significantly reduced anxiety-like behavior in adult zebrafish subjected to alcohol withdrawal. Together with our earlier findings from the light & dark test (Figure 3), these suggest that MIC-1 holds potential as therapeutic agents for anxiety management.

### 3.9. Molecular Docking

#### 3.9.1. Simulation of MIC-1 with the GABA_A_ Receptor

**Figure 6:**
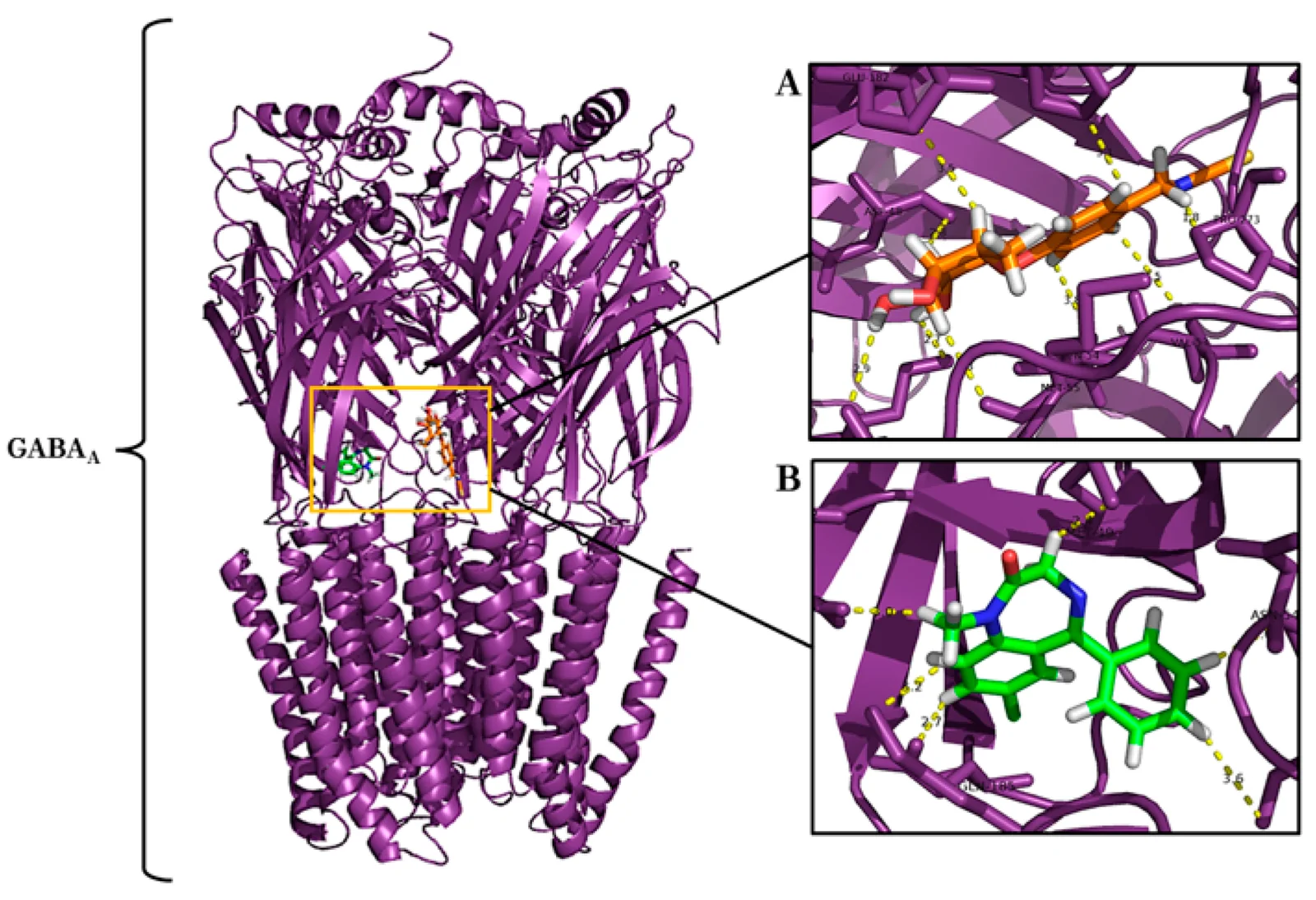
Molecular docking complexes between the GABA_A_ receptor (PDB ID: 4COF; magenta cartoon) and ligands MIC-1 (Stick representation of the structure: C in gold, H in white, N in blue, and O in red) and diazepam (Stick representation of the structure: C in green, H in white, N in blue, and O in red). (**A**) Binding site of MIC-1 on the GABA_A_ receptor, highlighting key interacting residues (Asp^48^, Val^53^, Asn^54^, Met^55^, Lys^102^, Ala^135^, Glu^182^, Pro^184^, and Pro^273^). Nine chemical interactions, including hydrogen bonds and van der Waals contacts, are shown as yellow dashed lines with distances ranging from 1.8 to 4.5 Å. (**B**) Superimposed complex showing MIC-1 (orange sticks) and diazepam (green sticks) bound at the same site on the GABA_A_ receptor.

To validate the potential interaction of MIC-1 with the GABA_A_ receptor, molecular docking simulations were performed (Figure 6). The most energetically favorable cluster exhibited a binding energy of −310.74 kcal, indicating a stronger interaction compared to the classical agonist diazepam, which presented a binding energy of −209.66 kcal.

**Figure 7:**
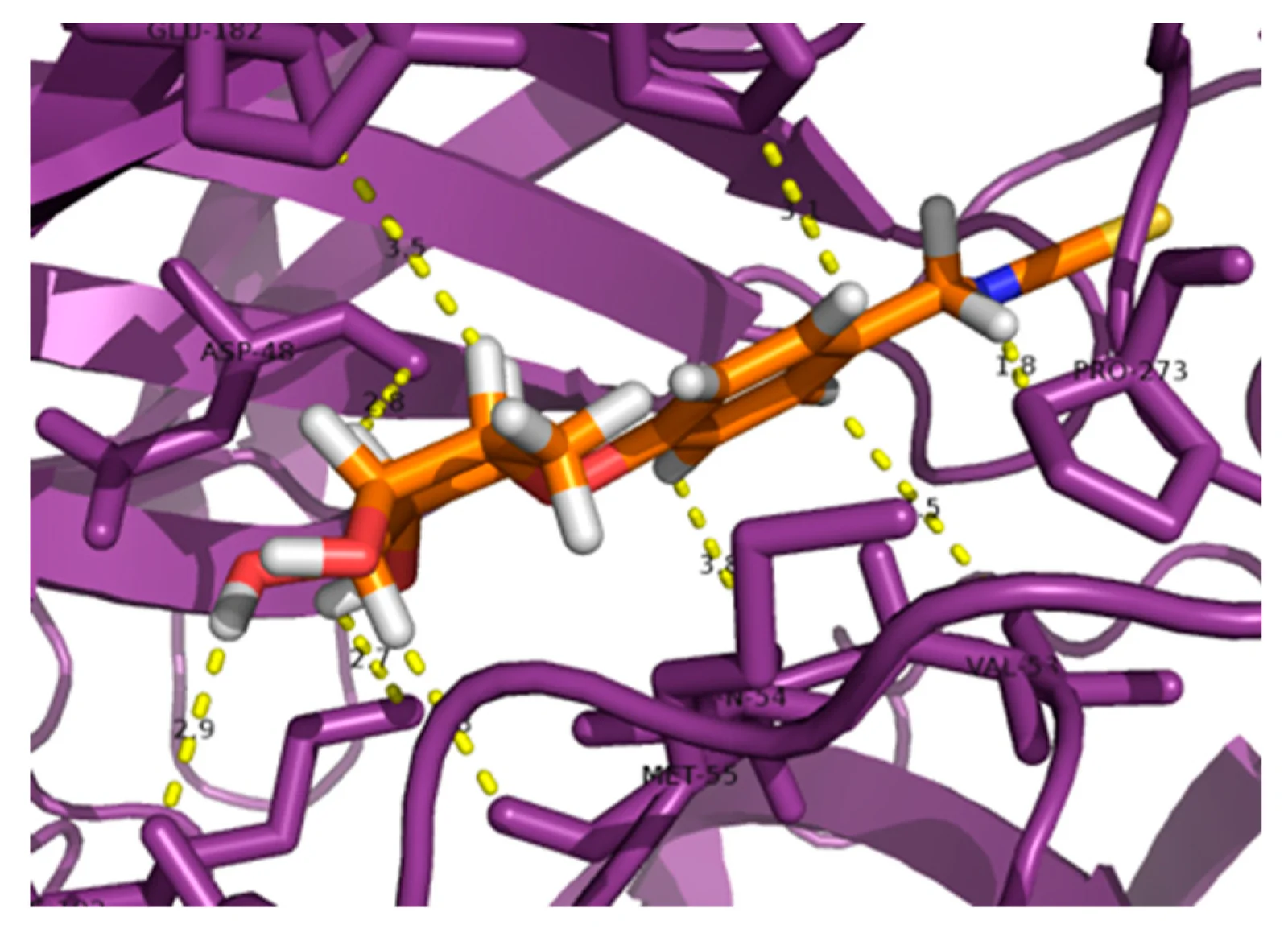
Binding site of MIC-1 (Stick representation of the structure: C in green, H in white, N in blue, and O in red) on the GABA_A_ receptor, showing nine chemical interactions (1.8 to 4.5 Å) with nine amino acid residues: Asp^48^, Val^53^, Asn^54^, Met^55^, Lys^102^, Ala^135^, Glu^182^, Pro^184^, and Pro^273^.

MIC-1 demonstrated high affinity for the central channel region of the receptor, interacting with amino acid residues within a domain characterized by secondary structural elements, including loops and β-sheets. MIC-1 established nine chemical interactions (hydrogen bonds and van der Waals forces) with the GABA_A_ receptor, involving amino acid residues Asp^48^, Val^53^, Asn^54^, Met^55^, Lys^102^, Ala^135^, Glu^182^, Pro^184^, and Pro^273^. These interactions ranged in distance from 1.8 to 4.5 Å (Figure 7).

**Figure 8:**
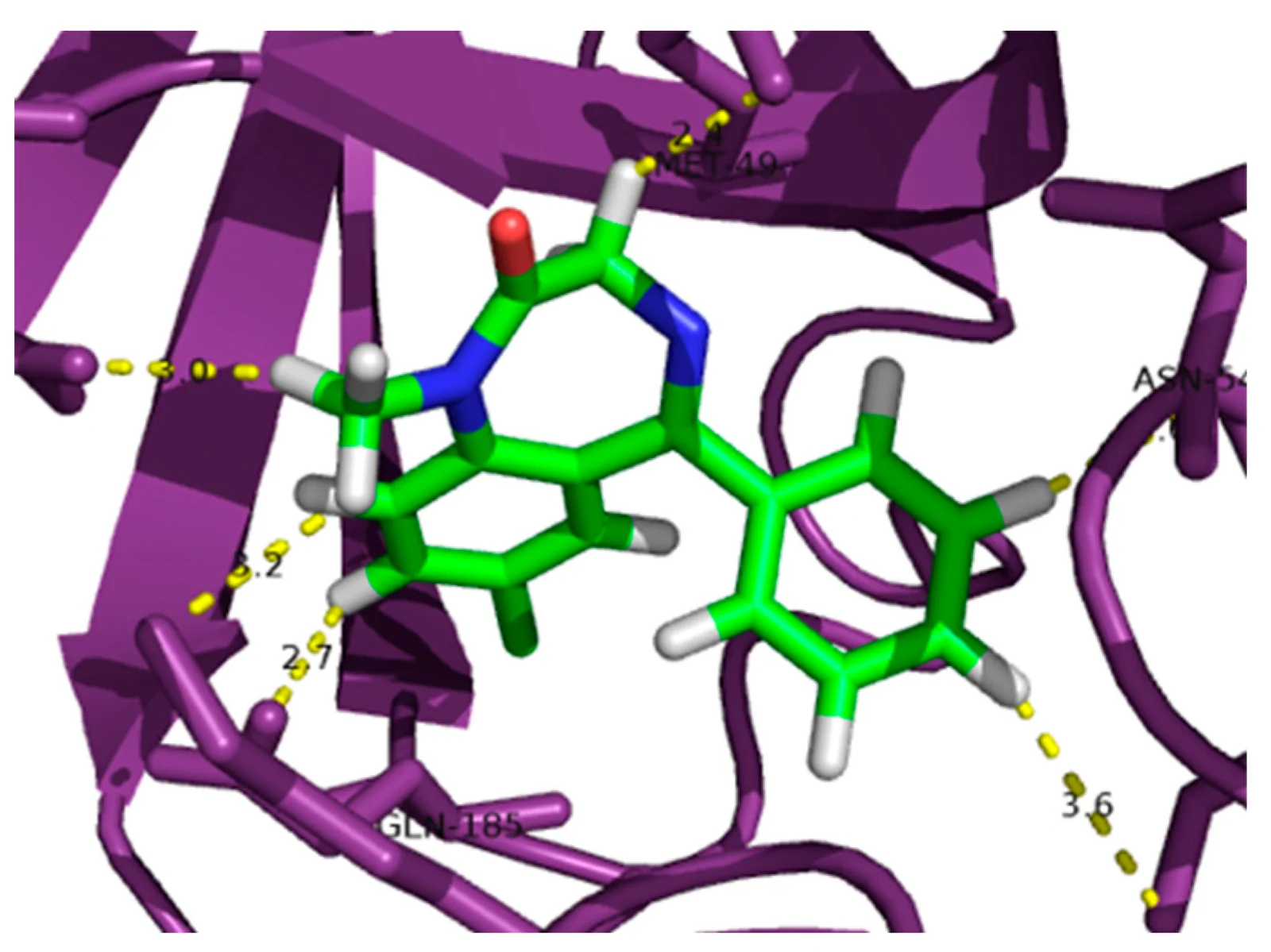
Binding site of diazepam (Stick representation of the structure: C in green, H in white, N in blue, and O in red) on the GABA_A_ receptor: six chemical interactions (2.4 to 3.6 Å) were observed, involving six amino acid residues of the receptor: Met^49^, Glu^52^, Asn^54^, Met^55^, Gln^185^, and Lys^274^.

Finally, it is worth noting that the complexation of diazepam (green) with the GABA_A_ receptor resulted in six chemical interactions (ranging from 2.4 to 3.6 Å) involving six amino acid residues: Met^49^, Glu^52^, Asn^54^, Met^55^, Gln^185^, and Lys^274^. The most energetically favorable cluster showed an energy loss of −209.66 kcal upon ligand binding (Figure 8).

#### 3.9.2. Molecular Docking of MIC-1 with the 5-HT_1B_ Receptor

**Figure 9:**
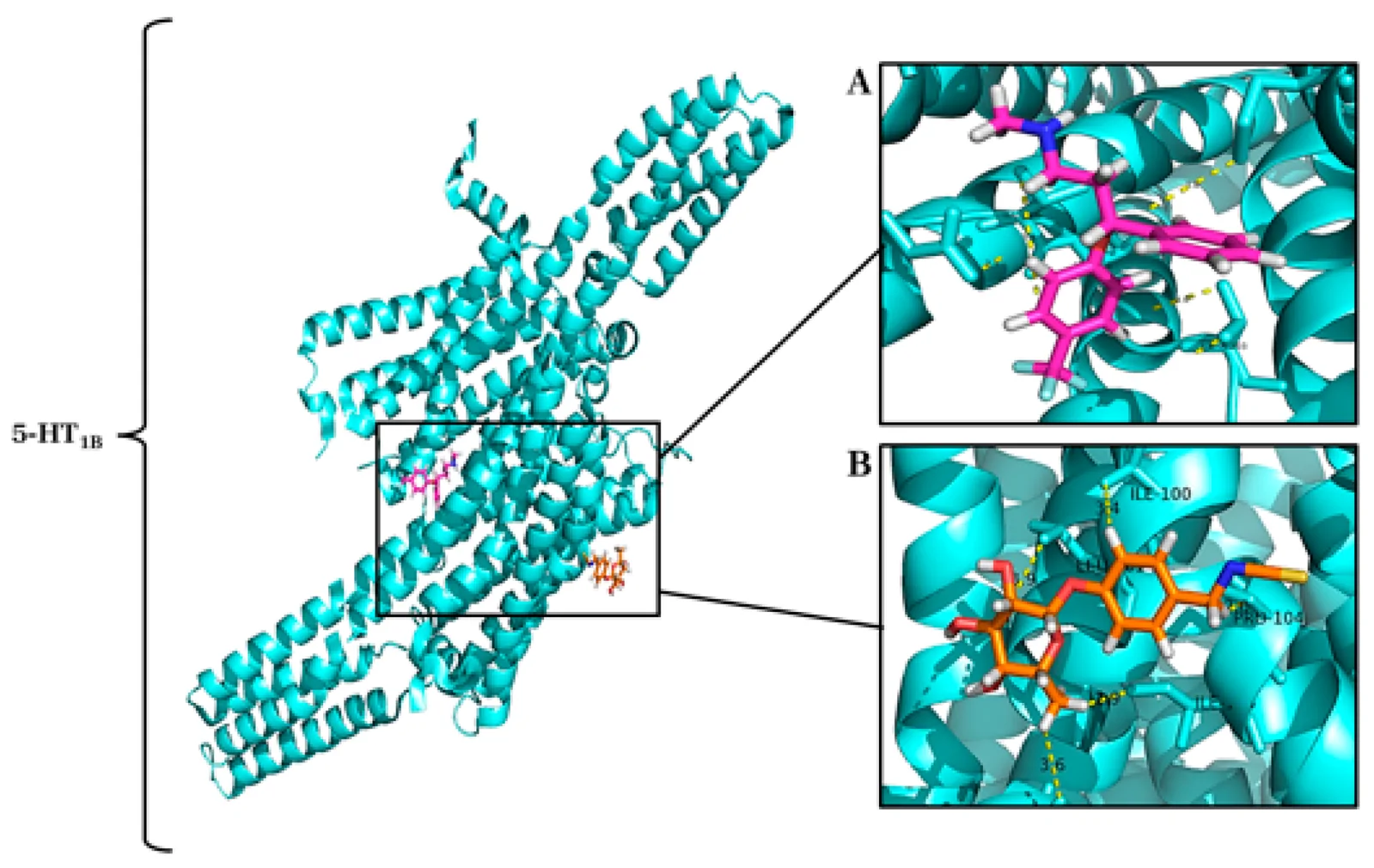
Complex formed between the 5-HT_1B_ receptor (PDB ID: 5V54) (cyan cartoon representation) and the ligands MIC-1 (Stick representation of the structure: C in gold, H in white, N in blue, and O in red) and fluoxetine (Stick representation of the structure: C in magenta, H in white, N in blue, and O in red): (**A**) Fluoxetine binding site, representing the classical antagonist site of the 5-HT_1B_ receptor. (**B**) MIC-1 binding site, demonstrating five chemical interactions (hydrogen bonds and hydrophobic interactions) involving five amino acid residues: Ile^100^, Leu^101^, Ile^105^, and Pro^104^, and Val^121^.

Regarding the 5-HT_1B_ receptor, the most energetically favorable cluster revealed an energy loss of −212.33 kcal upon MIC-1 binding, indicating a relevant interaction compared to the binding energy of the classical antagonist fluoxetine (−313.84 kcal). It is likely that a primary and secondary binding site on the 5-HT_1B_ receptor exists for MIC-1, as MIC-1 exhibited affinity and specificity for a binding site distinct from that of fluoxetine, a known modulator of 5-HT_1B_ receptor binding sites (Figure 9).

**Figure 10:**
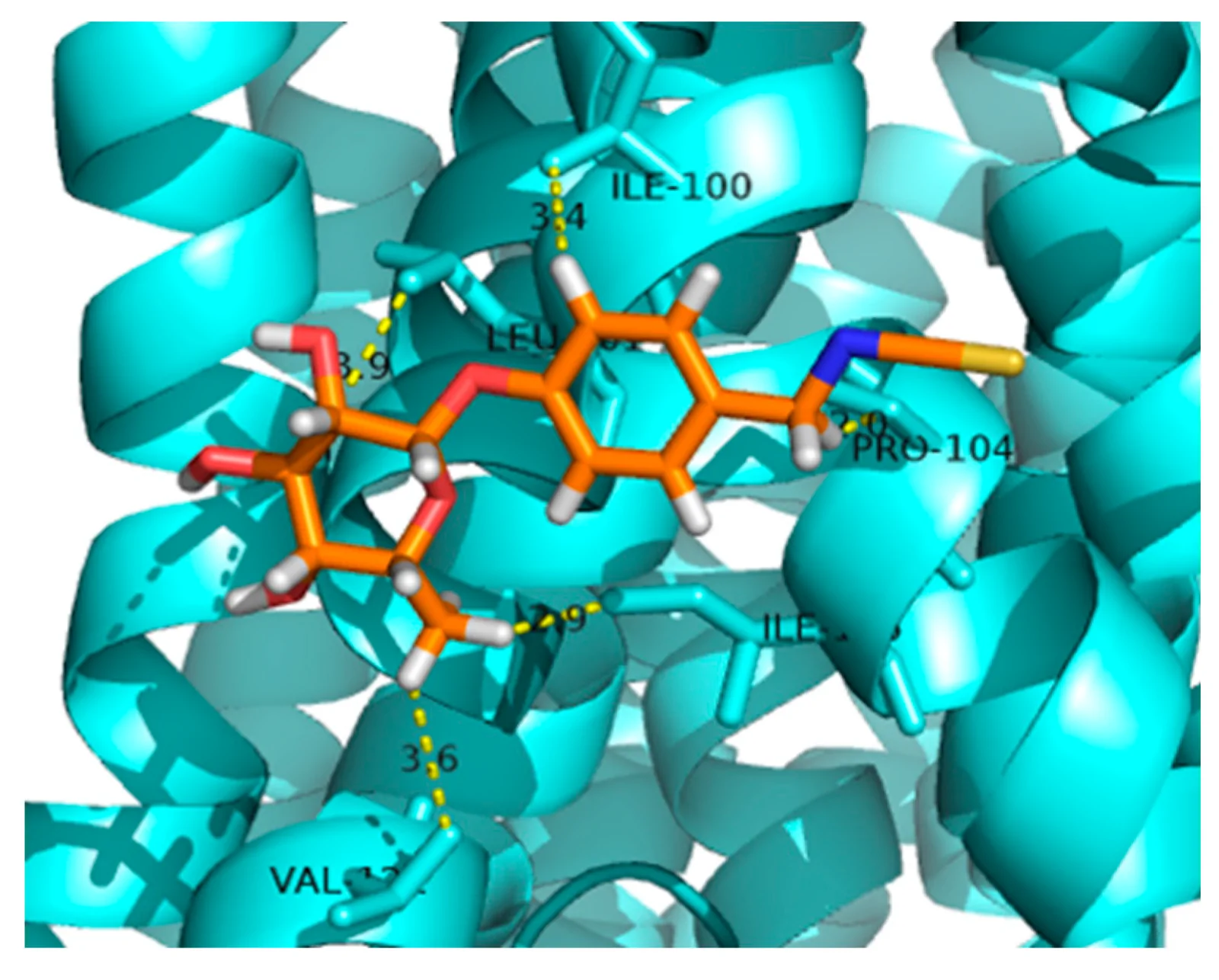
Binding site of MIC-1 (Stick representation of the structure: C in gold, H in white, N in blue, and O in red) on the 5-HT_1B_ receptor: evidence of five chemical interactions (2.0 to 3.9 Å) involving five amino acid residues of the receptor: Ile^100^, Leu^101^, Ile^105^, and Pro^104^, and Val^121^.

The interaction between MIC-1 and the 5-HT_1B_ receptor involved five chemical bonds (ranging from 2.0 to 3.9 Å) formed through the recruitment of five amino acid residues: Ile^100^, Leu^101^, Ile^105^, and Pro^104^, and Val^121^ (Figure 10).

**Figure 11:**
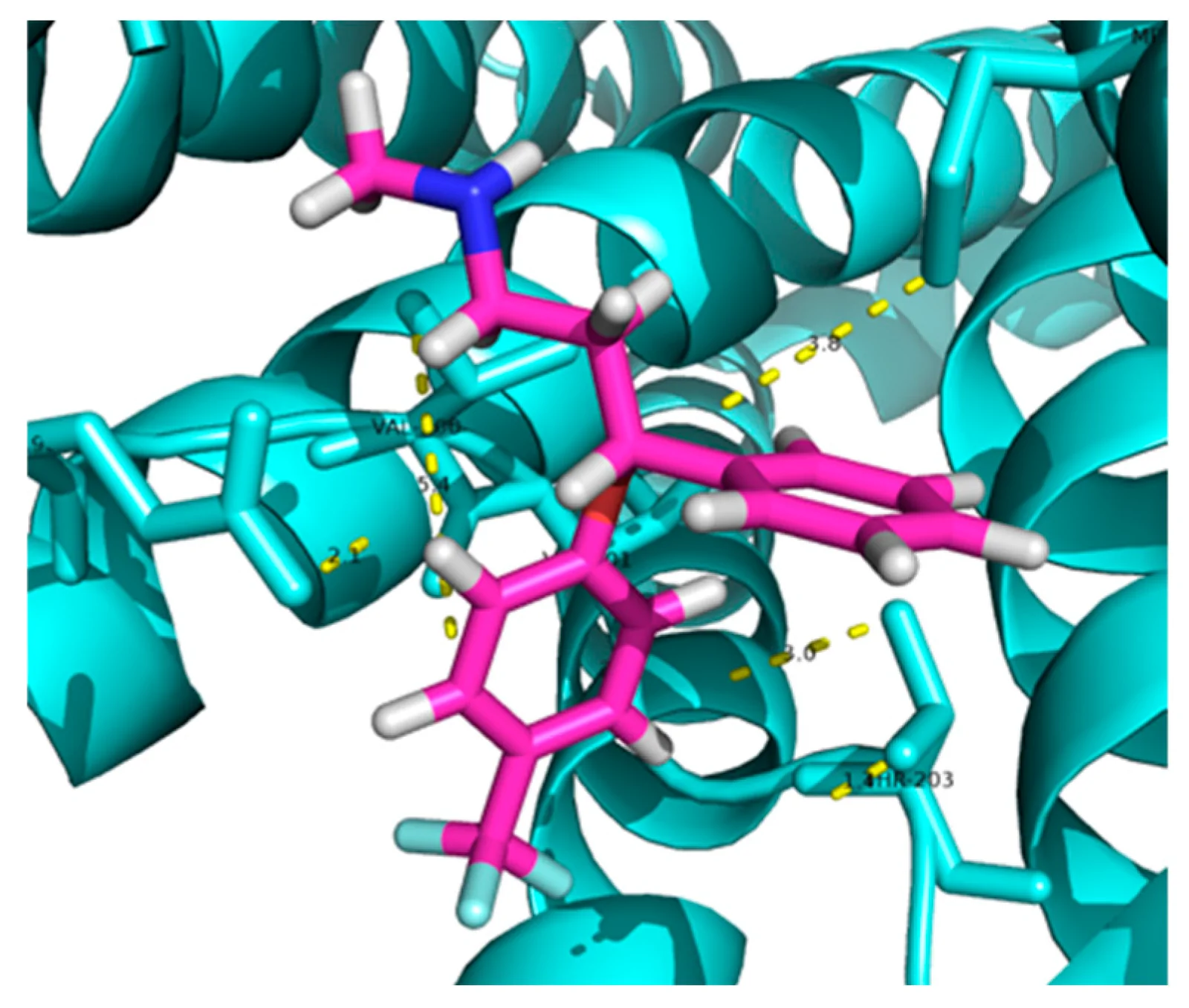
Binding site of fluoxetine (Stick representation of the structure: C in magenta, H in white, N in blue, and O in red) on the 5-HT_1B_ receptor: six chemical interactions (1.4 to 5.4 Å) were observed, involving five amino acid residues of the receptor: Glu^198^, Val^201^, Val^200^, Thr^203^, and Met^337^.

It is worth mentioning that during the complexation of fluoxetine (magenta) with the 5-HT_1B_ receptor, six chemical interactions (ranging from 1.4 to 5.4 Å) were identified, involving five amino acid residues of the receptor: Glu^198^, Val^201^, Val^200^, Thr^203^, and Met^337^. The most energetically favorable binding cluster demonstrated a total energy loss of −313.84 kcal following ligand complexation, indicating strong binding affinity (Figure 11).

#### 3.9.3. Molecular Docking of MIC-1 with the 5-HT_2A/C_ Receptor

**Figure 12:**
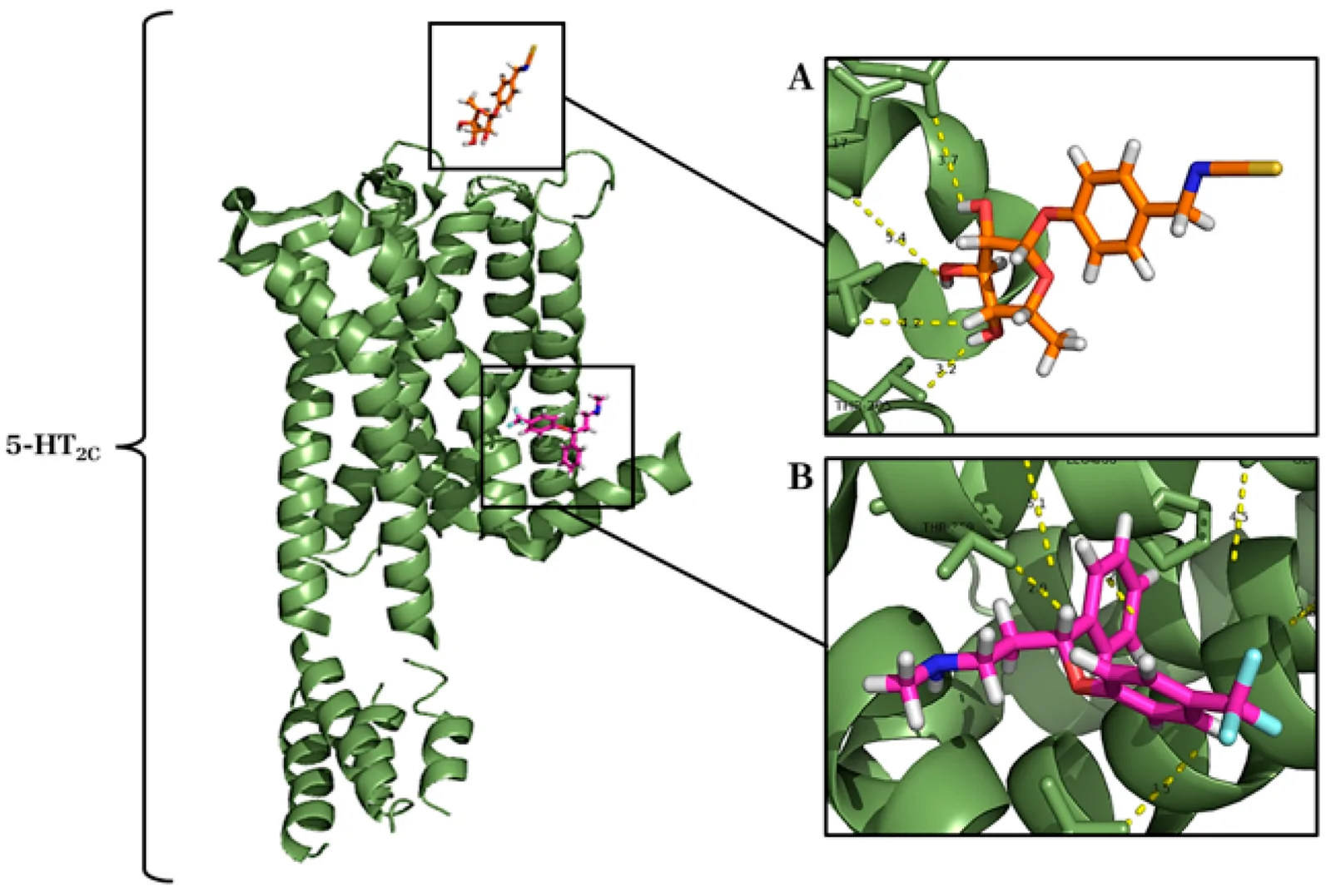
Complex formed between the 5-HT_2A/C_ receptor (PDB ID: 6BQH) (green cartoon representation) and the ligands MIC-1 (Stick representation of the structure: C in gold, H in white, N in blue, and O in red) and fluoxetine (Stick representation of the structure: C in pink, H in white, N in blue, and O in red): (**A**) Binding site of MIC-1, showing 4 chemical interactions (hydrogen bonds and hydrophobic contacts) with 4 amino acid residues: Gln^53^, Asp^117^, Val^119^, and Thr^205^. (**B**) Binding site of fluoxetine (classic antagonist) on the receptor.

The complex formed between MIC-1 and the 5-HT_2A/C_ receptors exhibited an energetically favorable cluster with an energy loss of −49.28 kcal following ligand complexation, indicating a relatively weak interaction compared to the classical antagonist fluoxetine, which showed an energy loss of −266.89 kcal. MIC-1 demonstrated affinity and specificity for a binding site distinct from that of fluoxetine, a direct-acting ligand of another 5-HT_2A/C_ receptor subtype, as illustrated in Figure 12. Despite the weaker interaction, MIC-1 formed multiple chemical bonds and recruited several amino acid residues within its binding site.

**Figure 13:**
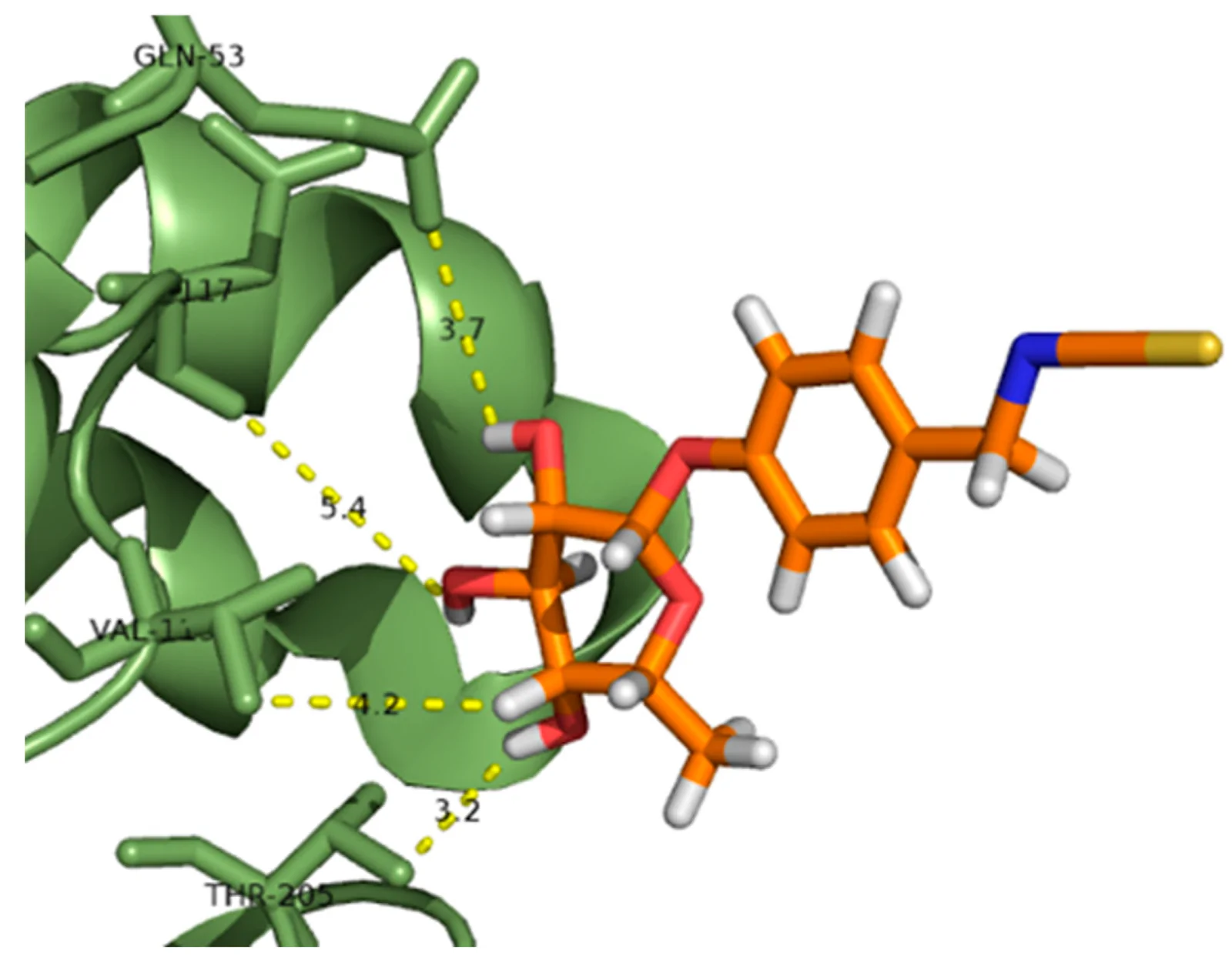
Binding site of MIC-1 (Stick representation of the structure: C in gold, H in white, N in blue, and O in red) on the 5-HT_2A/C_ receptor: evidence of four chemical interactions (3.2 to 5.4 Å) with four amino acid residues of the receptor (Gln^53^, Asp^117^, Val^119^, and Thr^205^).

The interaction of MIC-1 with the 5-HT_2A/C_ receptor involved the recruitment of four amino acid residues: Gln^53^, Asp^117^, Val^119^, and Thr^205^. Four chemical bonds ranging from 3.2 to 5.4 Å were formed between MIC-1 and these residues, as illustrated in Figure 13.

**Figure 14:**
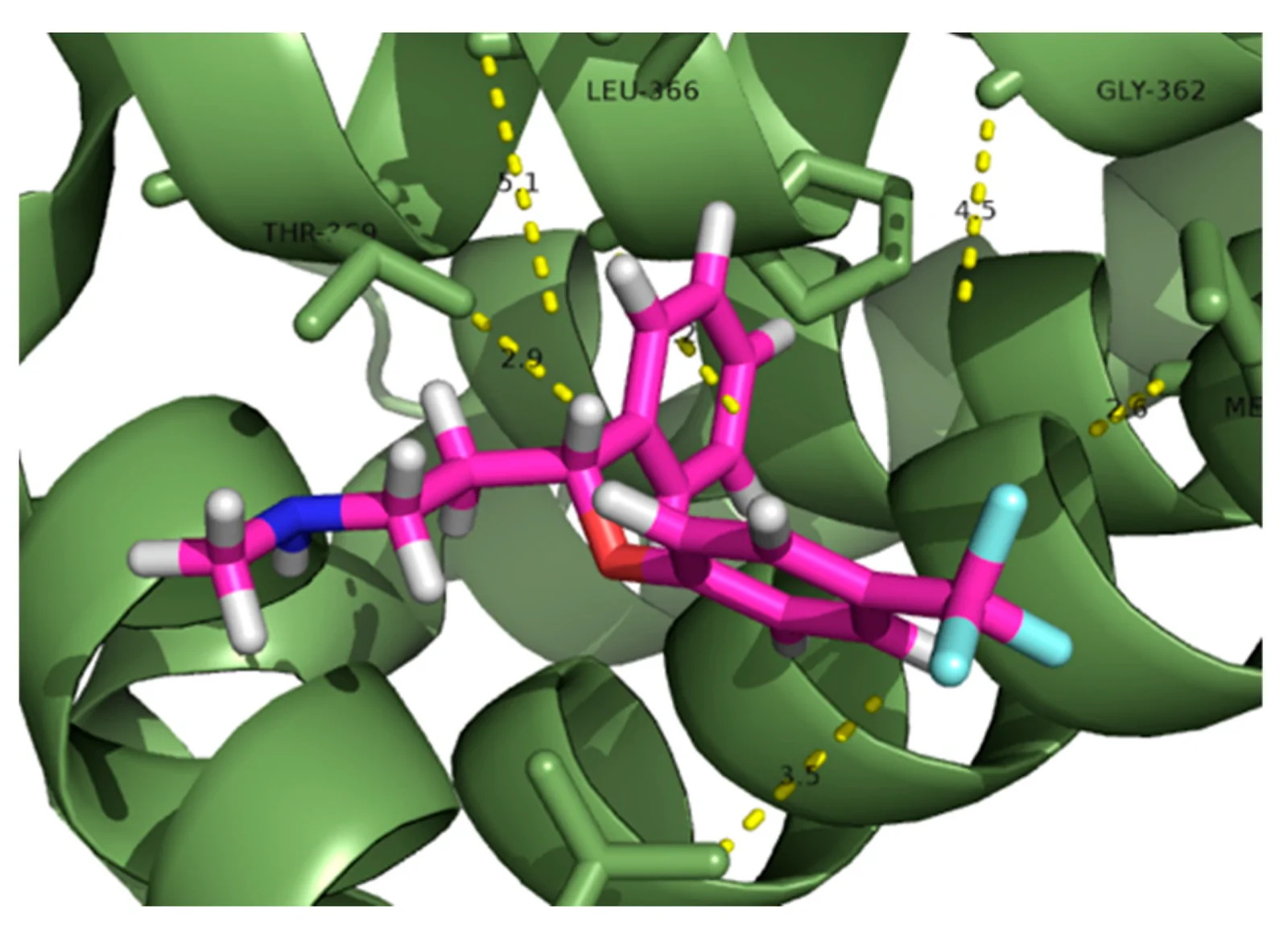
Binding site of fluoxetine (Stick representation of the structure: C in magenta, H in white, N in blue, and O in red) on the 5-HT_2A/C_ receptor: evidence of 6 chemical interactions (2.6 to 5.1 Å) with 6 amino acid residues of the receptor (Met^66^, Gly^362^, Pro^365^, Leu^366^, Thr^369^, and Leu^383^).

It is important to mention that in the complexation of fluoxetine (pink) with the 5-HT_2C_ receptor, six chemical bonds (2.6 to 5.1 Å) were observed involving six amino acid residues of the receptor: Met^66^, Gly^362^, Pro^365^, Leu^366^, Thr^369^, and Leu^383^. The most energetically favorable cluster exhibited an energy loss of −266.89 kcal after ligand complexation (Figure 14).

#### 3.9.4. Molecular Docking of MIC-1 with 5HT_3A/B_ Receptor

**Figure 15:**
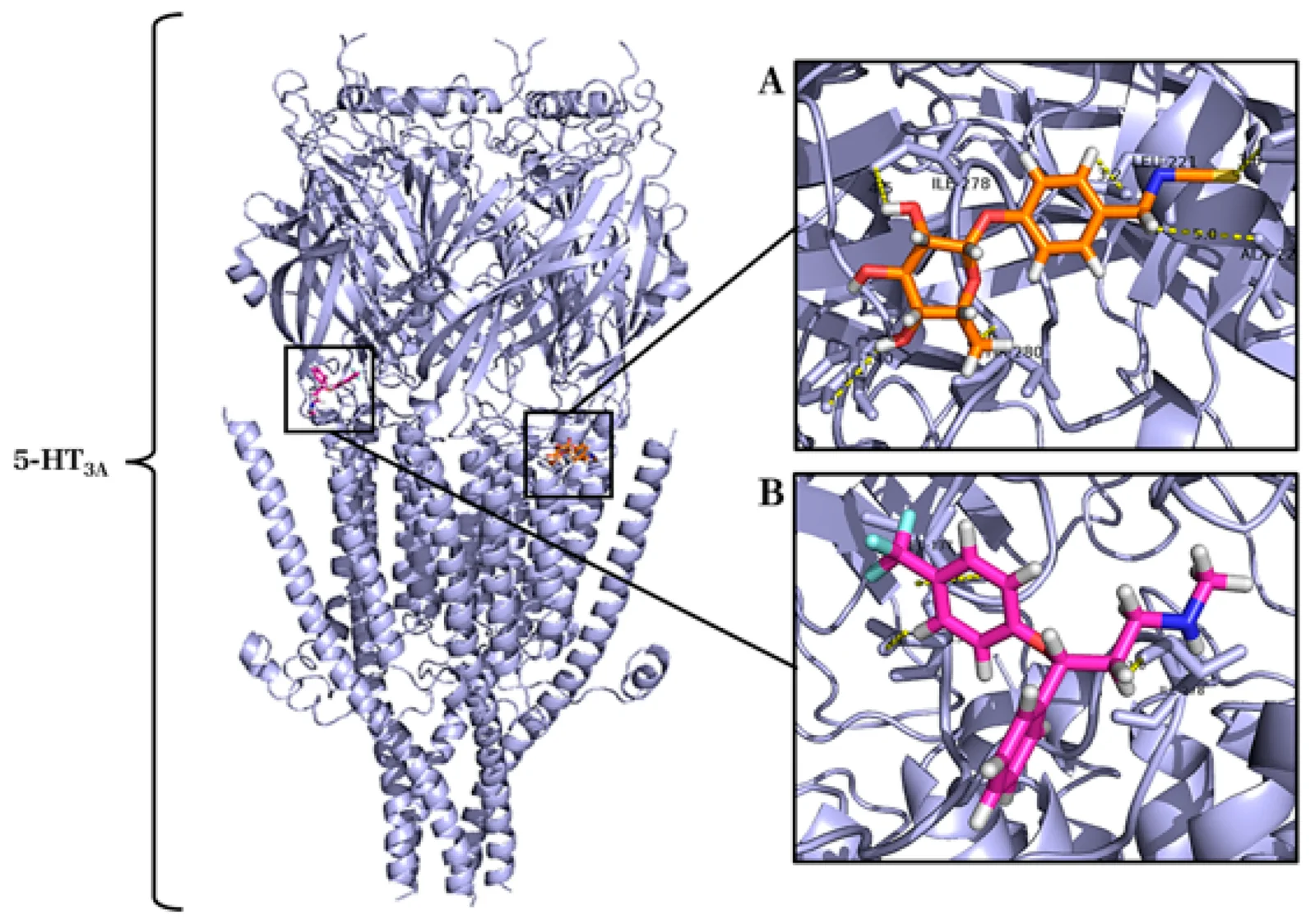
Complex formed between the 5-HT_3A/B_ receptor (PDB ID: 6W1Y) (light blue cartoon representation) and the ligands MIC-1 (Stick representation of the structure: C in gold, H in white, N in blue, and O in red) and fluoxetine (Stick representation of the structure: C in magenta, H in white, N in blue, and O in red): (**A**) Binding site of MIC-1, highlighting 6 chemical interactions (hydrogen bonds and hydrophobic interactions) involving 6 amino acid residues: Leu^221^, Ala^224^, Tyr^140^, Trp^456^, Ile^278^, and Thr^280^. (**B**) Binding site of MIC-1 located near the fluoxetine binding site.

The complex formed between MIC-1 and the 5-HT_3A/B_ receptor showed that the most energetically favorable cluster presented an energy loss of −181.23 kcal after ligand complexation, indicating a relevant interaction compared to the binding energy of the classical antagonist fluoxetine (−240.95 kcal). MIC-1 exhibited affinity and specificity for a binding site distinct from that of fluoxetine, which interacts with known binding sites of the 5-HT_3A/B_ receptor. This interaction involved several chemical bonds and the recruitment of multiple amino acid residues, as illustrated in Figure 15.

**Figure 16:**
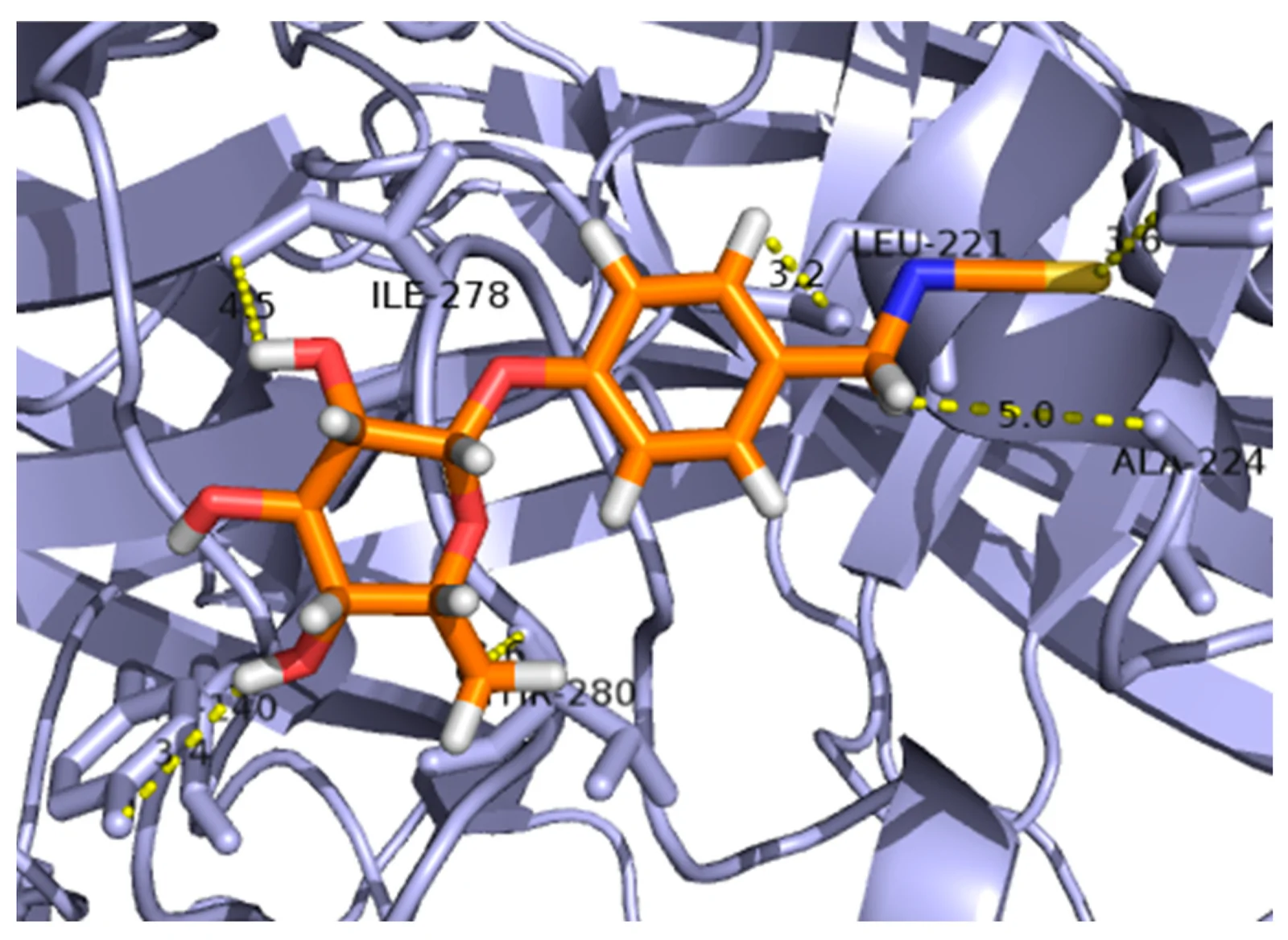
Binding site of MIC-1 (Stick representation of the structure: C in gold, H in white, N in blue, and O in red) on the 5-HT_3A/B_ receptor: showing six chemical interactions (3.2 to 5.0 Å) with six amino acid residues of the receptor (Leu^221^, Ala^224^, Tyr^140^, Trp^456^, Ile^278^, and Thr^280^).

The interaction of MIC-1 with the 5-HT_3A/B_ receptor involved the recruitment of six amino acid residues: Leu^221^, Ala^224^, Tyr^140^, Trp^456^, Ile^278^, and Thr^280^. Six chemical bonds, ranging from 3.2 to 5.0 Å, were established between MIC-1 and these residues, as illustrated in Figure 16.

**Figure 17:**
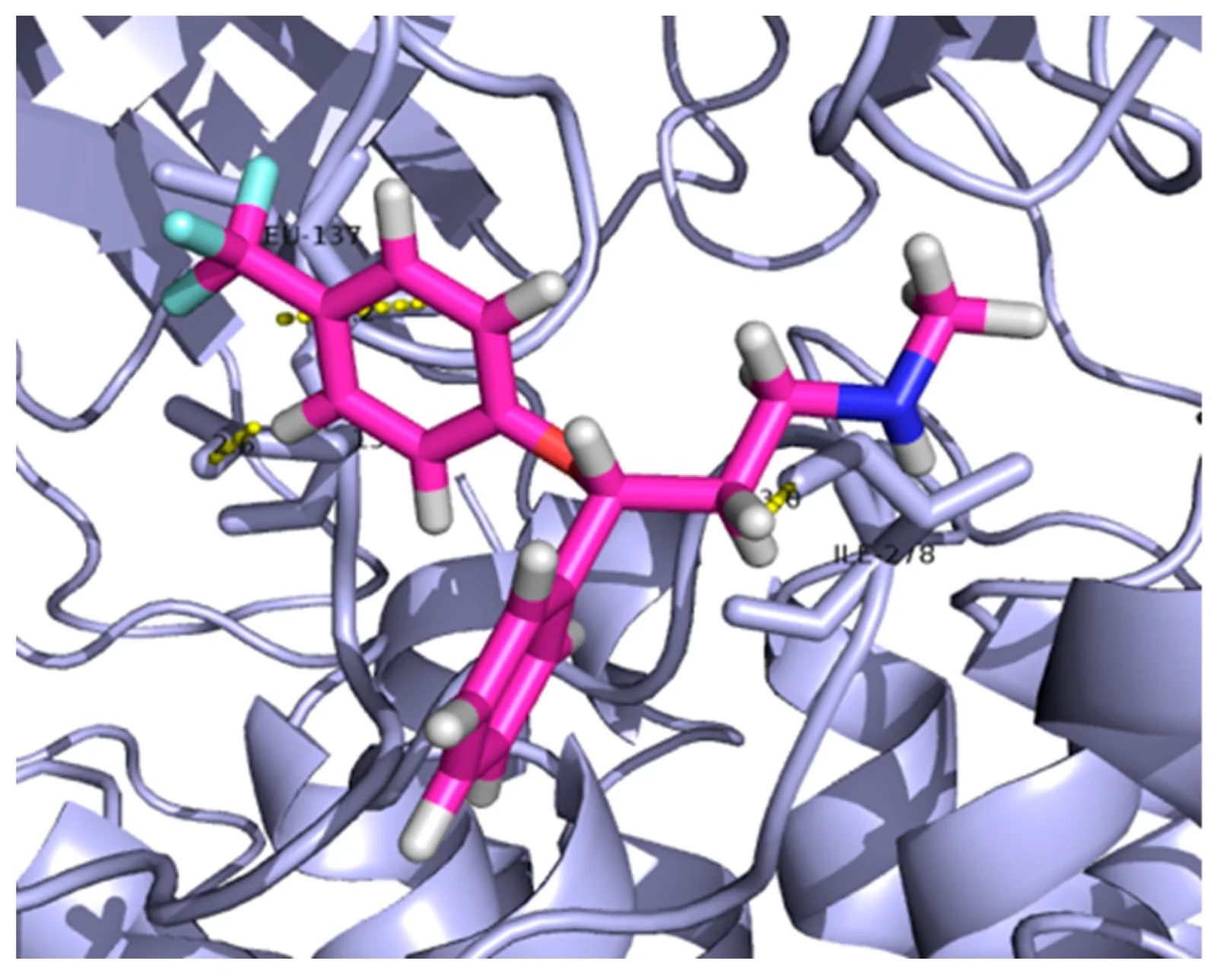
Binding site of fluoxetine (Stick representation of the structure: C in magenta, H in white, N in blue, and O in red) on the 5-HT_3A/B_ receptor: evidence of three chemical interactions (2.0 to 5.2 Å) involving three amino acid residues of the receptor (Leu^137^, Asp^138^, and Ile^278^).

It is worth mentioning that in the complexation of fluoxetine (magenta) with the 5-HT_3A/B_ receptor, three chemical bonds (2.0 to 5.2 Å) were observed involving three amino acid residues of the receptor: Leu^137^, Asp^138^, and Ile^278^. The most energetically favorable cluster showed an energy loss of –240.95 kcal after ligand complexation, as illustrated in Figure 17.

## 4. Discussion

The present study is pioneering in elucidating the anxiolytic and anticonvulsant mechanisms of MIC-1 isolated from *M*. *oleifera* hydroalcoholic seed extract. Toxicological evaluation is essential for informed clinical and preclinical decision-making [27], as it determines the therapeutic applicability of candidate compounds [28]. In this study, MIC-1 demonstrated a favorable safety profile, with no mortality observed in adult zebrafish (*D. rerio*) during the 96-h exposure period. Locomotor activity analysis in zebrafish is a widely accepted behavioral parameter for assessing the central nervous system effects of pharmacological agents and detecting potential motor impairments [20]. The administration of MIC-1 did not alter zebrafish locomotion, suggesting that MIC-1 may affect the central nervous system without changes in motor function.

Epileptic seizures, which may escalate to status epilepticus, present significant therapeutic challenges, particularly in settings lacking continuous electroencephalogram monitoring. Although corticosteroids are increasingly being explored as adjunctive treatments for epilepsy, their efficacy and mechanisms remain insufficiently validated [29]. This gap in evidence underscores the need for novel therapeutic approaches, including the off-label use of steroidal anti-inflammatory agents [30]. Emerging studies have highlighted the neuropharmacological potential of *M. oleifera* extracts, suggesting anxiolytic and anticonvulsant effects, possibly mediated through modulation of the GABAergic system [31,32,33]. Based on this evidence, the present study aimed to evaluate the neurobiological effects of phytocompound MIC-1, isolated from an *M. oleifera* seed extract, through a series of behavioral, pharmacological, and In silico tests, with a focus on elucidating their mechanisms of action.

In this study, MIC-1 did not exhibit sedative effects in adult zebrafish. However, MIC-1 demonstrated significant anticonvulsant activity, which appears to be mediated through interaction with the GABA_A_ receptor. Notably, MIC-1 exhibited this effect at low doses, highlighting its potent pharmacological potential. The involvement of the GABAergic system in this activity was confirmed using flumazenil, a selective GABA_A_ receptor antagonist, which effectively reversed the anticonvulsant effects of MIC-1. These findings suggest that the anticonvulsant action of MIC-1 is GABA_A_ receptor-dependent.

The light & dark test in zebrafish is a well-established behavioral assay used to evaluate anxiety-related responses based on the species’ natural preference for dark environments and an aversion to brightly lit areas [22]. An anxiolytic-like effect is typically indicated by increased time spent in the light zone and a higher number of transitions between light and dark zones. These behavioral changes suggest a reduction in anxiety-like behavior due to decreased avoidance of the aversive (light) environment. Therefore, this test is a valuable tool for screening compounds with potential anxiolytic activity and for exploring the underlying neurobiological mechanisms of anxiety [23]. In the present study, MIC-1 demonstrated anxiolytic-like effects in the light & dark test.

We applied the same experimental approach (light & dark test) to assess the potential effects of MIC-1 on anxiety-like behavior induced by alcohol withdrawal in zebrafish. Alcohol at low doses can produce a calming effect by attenuating anxiety and stress; however, alcohol at higher doses induces sedation and significant behavioral changes in humans [34]. Chronic and excessive alcohol consumption may lead to the development of alcohol withdrawal syndrome, characterized by persistent symptoms such as tremors, seizures, hallucinations, autonomic dysregulation, and heightened anxiety [35]. Alcohol, a central nervous system depressant, initially produces anxiolytic effects by enhancing the activity of the neurotransmitter GABA. However, prolonged and excessive alcohol use increases oxidative stress and cellular damage, which can lead to heightened stress and anxiety, thereby contributing to a cycle of compulsive consumption and worsening the anxiety symptoms associated with alcohol withdrawal syndrome [36].

MIC-1 also effectively reduced anxiety-like behavior in the alcohol withdrawal model of anxiety, with effects comparable to those observed for diazepam. These findings reinforce the therapeutic potential of MIC-1 in managing withdrawal-induced anxiety and further validate the use of zebrafish as a reliable model for evaluating anxiety associated with alcohol withdrawal, as previously reported [16,37].

The GABAergic system is responsible for the synthesis and action of gamma-aminobutyric acid (GABA), the primary inhibitory neurotransmitter in the central nervous system. Activation of GABA_A_ receptors induces neuronal hyperpolarization, thereby reducing excitability and producing calming and anxiolytic effects [38]. In this study, MIC-1 exhibited anxiolytic-like effects comparable to those of diazepam, a well-established GABA_A_ receptor agonist. Furthermore, the reversal of these effects by flumazenil, a selective GABA_A_ receptor antagonist, confirms that the anxiolytic-like activity of MIC-1 is mediated through the GABA_A_ receptor. These findings are consistent with previous reports suggesting GABAergic involvement in the effects of *M. oleifera* extracts [31,32,33]. The present study advances the current understanding by detailing the specific neuropharmacological pathways through which MIC-1 exerts its effects on the central nervous system.

Given that MIC-1 exhibited anxiolytic-like effects, we also examined the effects of MIC-1 on the serotonergic system (5-HT_1B_, 5-HT_2A/C_, and 5-HT_3A/3B_ receptor subtypes). Serotonin (5-HT) is a key neurotransmitter involved in the regulation of mood, appetite, anxiety, sleep, and various other central functions. The 5-HT_1B_, 5-HT_2A/C_, and 5-HT_3A/3B_ receptors, which are widely expressed in the central nervous system, play critical roles in modulating serotonergic signaling. Evidence suggests that activation of these receptor subtypes is often associated with anxiogenic responses, whereas their inhibition may result in anxiolytic effects [39,40].

In this study, the lack of reversal of the anxiolytic-like effects of MIC-1 by pre-treatment with cyproheptadine, a selective 5-HT_2A/C_ antagonist, suggests that MIC-1’s anxiolytic effects are not mediated through the serotonergic 5-HT_2A/C_ receptor. In contrast, the significant reversal of the anxiolytic-like effects by pre-treatment with pizotifen (a 5-HT_1B_ and 5-HT_2A/C_ antagonist) and granisetron (a 5-HT_3A/3B_ antagonist) indicates that the serotonergic 5-HT_1B_, 5-HT_2A/C_, and 5-HT_3A/3B_ receptors are likely involved in MIC-1’s mechanism of action.

Molecular docking improves the precision of pharmacological analyses by elucidating complex molecular interactions based on the structural complementarity between drugs and target proteins. By identifying compounds with similar characteristics, it helps reveal shared biological pathways. This technique is essential for drug validation and repositioning, thereby optimizing the discovery and development of new medications [41]. To add robustness to the experimental data obtained from the animal model discussed above, we employed bioinformatics tools.

To validate the involvement of specific systems, molecular docking simulations were performed with MIC-1. The docking results with the GABA_A_ receptor revealed that MIC-1 exhibited strong affinity for the center of the channel, forming an energetically favorable cluster superior to that of diazepam, suggesting a stronger and more stable interaction. MIC-1 interacted with amino acid residues located in a region characterized by loops and β-sheets, showing affinity and binding patterns similar to diazepam. In the case of the 5-HT_1B_ receptor, MIC-1 presented a relevant energetic cluster compared to fluoxetine, binding specifically to a site distinct from that of fluoxetine. This finding suggests the existence of both primary and secondary binding sites on the 5-HT_1B_ receptor for MIC-1. For the 5-HT_2A/C_ receptor, MIC-1 showed a much weaker energetic cluster relative to fluoxetine and bound to a site distant from fluoxetine’s binding region. Lastly, with the 5-HT_3A/B_ receptor, MIC-1 demonstrated a relevant energetic cluster compared to fluoxetine Also, MIC-1 showed affinity and specificity for a binding site distinct from that of fluoxetine, which is known to interact with the 5-HT_3A/B_ receptor binding sites.

The discovery of molecules, along with their physicochemical properties and molecular targets, holds great promise but also presents significant challenges due to the vast variability in molecular quantity, structure, interactions, and dynamics. Consequently, computational mapping through simulations, combined with experimental testing, plays a crucial role in drug discovery and development [42]. Therefore, evidence derived from molecular modeling offers a solid scientific foundation for further investigations and supports the potential development of MIC-1 as a therapeutic agent for anxiety and seizure disorders.

Several studies have demonstrated that other isothiocyanates may exhibit neuroprotective, neuromodulatory, anxiolytic, antidepressant, and anticonvulsant properties, highlighting their potential as therapeutic agents for neurological comorbidities [43,44,45]. The development of novel therapeutic strategies for anxiety and seizure disorders using phytochemicals is essential to provide effective and safer interventions. Moreover, understanding MIC-1’s neurochemical mechanisms provide valuable insights into the involvement of different neurotransmitter systems in the brain.

## 5. Conclusions

The results of this study indicate that *Moringa* isothiocyanate-1 (MIC-1), isolated from a hydroalcoholic extract of *M. oleifera* seeds, exhibits potential for neurological modulation by reducing anxiety-like and convulsive behaviors without causing sedation and mortality in adult zebrafish (*D. rerio*). Also, MIC-1 effectively mitigated anxiety induced by alcohol withdrawal, likely through MIC-1’s interactions with the GABAergic system. These findings were supported by In silico analyses revealing the specific binding of MIC-1 to the GABA_A_ receptor. Overall, this study underscores the therapeutic potential of MIC-1 for anxiety-related disorders and warrants further studies to elucidate its mechanisms of action and potential clinical applications.

## Data Availability

The original contributions presented in this study are included in the article. Further inquiries can be directed to the corresponding author(s).
